# Δ133p53 isoform enhances TLR4 function to promote tumor growth

**DOI:** 10.1093/carcin/bgaf051

**Published:** 2025-08-29

**Authors:** Sasini Polwatta Lekamlage, Alexandra N Boix De Jesus, Adriana Machado Saraiva, Catherine Drummond, Harrison Dolan, Francesc March de Ribot, Janice A Royds, Sunali Mehta, Antony W Braithwaite, Noelyn A Hung, Tania L Slatter

**Affiliations:** Department of Pathology, Dunedin School of Medicine, University of Otago, Dunedin 9016, New Zealand; Department of Pathology, Dunedin School of Medicine, University of Otago, Dunedin 9016, New Zealand; Department of Surgery, Division of Surgical Oncology, UCLA, Los Angeles, CA 90024, United States; Department of Pathology, Dunedin School of Medicine, University of Otago, Dunedin 9016, New Zealand; Department of Medical Laboratory Science, School of Pharmacy, University of Otago, Dunedin 9016, New Zealand; Department of Pathology, Dunedin School of Medicine, University of Otago, Dunedin 9016, New Zealand; Maurice Wilkins Centre for Molecular Biodiscovery, Auckland 1010, New Zealand; Department of Medical Laboratory Science, School of Pharmacy, University of Otago, Dunedin 9016, New Zealand; Department of Medicine, Dunedin School of Medicine, University of Otago, Dunedin 9016, New Zealand; Department of Medicine, Dunedin School of Medicine, University of Otago, Dunedin 9016, New Zealand; Department of Pathology, Dunedin School of Medicine, University of Otago, Dunedin 9016, New Zealand; Department of Pathology, Dunedin School of Medicine, University of Otago, Dunedin 9016, New Zealand; Maurice Wilkins Centre for Molecular Biodiscovery, Auckland 1010, New Zealand; Department of Pathology, Dunedin School of Medicine, University of Otago, Dunedin 9016, New Zealand; Maurice Wilkins Centre for Molecular Biodiscovery, Auckland 1010, New Zealand; Department of Pathology, Dunedin School of Medicine, University of Otago, Dunedin 9016, New Zealand; Department of Pathology, Dunedin School of Medicine, University of Otago, Dunedin 9016, New Zealand; Department of Medical Laboratory Science, School of Pharmacy, University of Otago, Dunedin 9016, New Zealand; Maurice Wilkins Centre for Molecular Biodiscovery, Auckland 1010, New Zealand

**Keywords:** cancer, **Δ**133p53, TLR4, API5, cell surface

## Abstract

Tumor protein 53 (TP53) acts as a tumor suppressor and is often mutated in cancer. Isoforms of TP53, such as the **Δ**133p53 family, can promote tumor growth and metastasis. Therefore, targeting **Δ**133p53 function may represent a new strategy for preventing tumor metastasis. To inform the identification of proteins to target in **Δ**133p53-expressing tumors, changes at the cell surface were characterized. Inhibition of cell surface trafficking in a mouse model syngrafted with tumors expressing proteins similar to **Δ**133p53 (**Δ**122p53) was associated with reduced tumor growth and metastasis. After confirming that changes at the cell surface were important for **Δ**133p53 tumor promotion, characterization of protein changes at the **Δ**133p53/**Δ**122p53 cell surface revealed increased expression of the toll-like receptor 4 (TLR4) and the TLR4 agonist, apoptosis inhibitor 5. Furthermore, inhibition of TLR4 was sufficient to reduce **Δ**122p53 tumor growth. Altogether, these results suggest a role for **Δ**133p53 in contributing to tumor progression by stimulating TLR4 function. Furthermore, targeting changes at the cell surface can reduce **Δ**133p53 tumor promotion.

## Graphical Abstract

**Graphical Abstract:**
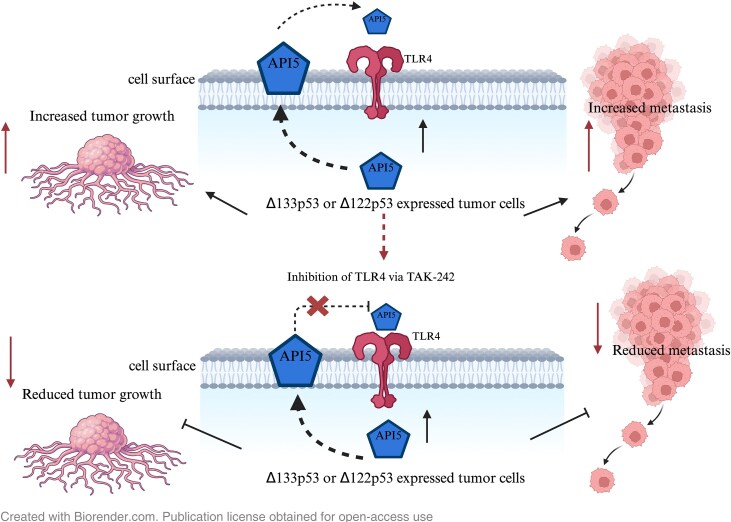

## 1. Introduction

Tumor protein 53 is central to cancer suppression, and p53 mutations are found in over half of all human cancers [1]. As most cancers are thought to have defective p53 function, alternatives to mutant p53 include the upregulation of p53 isoforms [2, 3]. Among the 12 p53 isoforms identified, the **Δ**133p53 isoforms (**Δ**133p53α, **Δ**133p53β, and **Δ**133p53γ) have been the most widely studied in cancer, with **Δ**133p53β being the most frequently reported isoform in cancers with poorer patient outcomes [4–9]. **Δ**133p53β is increased in metastases and tumors with increased infiltration of tumor-associated macrophages (TAMs) and immunosuppressive T cells [5, 6, 10, 11]. These associations with **Δ**133p53 isoforms, particularly **Δ**133p53β, suggest that **Δ**133p53 isoforms contribute to the tumor and promote tumor progression.

Alterations to the cell surface with **Δ**133p53 expression offer one explanation for tumor-promoting properties of **Δ**133p53. Identifying the altered proteins on the cell surface may provide potential targets for future therapeutic strategies. Evidence supporting a role for **Δ**133p53 at the tumor microenvironment interface exists with multiple cancer-promoting proteins having established cell surface roles that are increased in **Δ**133p53 cells or in cells expressing a murine protein similar to **Δ**133p53α called **Δ**122p53. Proteins increased at the cell surface of **Δ**133p53- or **Δ**122p53-expressing cells include programed death-ligand 1 (PDL1), MET proto-oncogene, receptor tyrosine kinase (c-MET), transferrin receptor (TFRC), epidermal growth factor receptor (EGFR), enolase 1 (ENO1), integrin subunit alpha 5 (ITGA5), and glucose transporter (GLUT) 1 and 4 [5, 6, 10, 12–14]. Consistent with the notion that proteins increased on **Δ**133p53 cells are suitable targets, TFRC upregulation and the associated increase in iron uptake were linked to greater susceptibility to ferroptosis following treatment with the small molecule R3L3 in lung cancer cell lines stably expressing **Δ**133p53 [10]. The increased expression of cancer-promoting proteins on the cell surface is reminiscent of mutant p53 function, in which increased levels of c-MET, TRFC, and EGFR are among the proteins with increased recycling on the cell surface [15–17]. In the context of mutant p53, increased levels of cell surface proteins promote invasion and metastasis. Whether **Δ**133p53-expressing cells have changes at the cell surface that contribute to the tumor microenvironment, and which could be exploited as therapeutic options for targeting aggressive cancers is uncertain.

Here, we explored the proteins altered on the surfaces of **Δ**133p53 and **Δ**122p53 cells in more detail and determined if the tumor-promoting properties of **Δ**133p53 were attributed to changes at the cell surface. We report that **Δ**133p53 and **Δ**122p53 are associated with many cancer-promoting proteins on the cell surface, which support tumor progression and metastasis. We observed a role for both an increase in toll-like receptor 4 (TLR4) and the TLR4 agonist apoptosis inhibitor 5 (API5) on the cell surface. Increased TLR4 signaling promoted **Δ**122p53 tumor growth and changes to the tumor microenvironment. These findings indicate that **Δ**133p53 contributes to the tumor microenvironment by upregulating cell surface receptors and secreted proteins, which can further activate receptor signaling in the tumor microenvironment. This study highlighted proteins that could be targeted to affect cells expressing **Δ**133p53.

## 2. Materials and methods

### 2.1. Tumor syngrafts

Four murine cells lines stably expressing **Δ**122p53, established in previous studies, or the corresponding controls [8, 18], were used. These included pancreatic ductal adenocarcinoma (PDAC) (**Δ**122p53PDAC and ControlPDAC), and B16F10 melanoma (**Δ**122p53B16F10 and ControlB16F10). Cells were syngrafted subcutaneously (2 × 10^6^ cells) into the left hind limb flank of wild-type C57BL/6J mice. Mice (15 per group) were euthanized when the tumor reached 1 cm in diameter or earlier if the tumor became ulcerated. When the tumor was first palpable, monensin (10 mg/kg), brefeldin A (10 mg/kg), a TLR4 inhibitor (TAK-242, 2.4 mg/kg Sigma Aldrich), or vehicle control (DMSO) was administered three times a week for 3 weeks [19]. To test whether monensin and brefeldin A treatment reduced the incidence of metastases with **Δ**122p53 tumors, separate cohorts of mice (*n* = 15 per group) were treated with monensin or brefeldin A for a continuous period of 10 days, commencing when tumors were first palpable. Mice were euthanized 24 h after treatment completion or as described above. Animal Ethical approval was approved by the Otago Animal Ethics Committee (references, AUP-20-53 and AUP-24-29).

### 2.2. Cell lines and culture

Stable human clonal cell lines expressing different **Δ**133p53 isoforms, derived from the H1299 cell line, were as previously described [6], and included those expressing **Δ**133p53α (**Δ**133p53α1, **Δ**133p53α2, and **Δ**133p53α8) or **Δ**133p53β (**Δ**133p53β5 and **Δ**133p53β10). The original H1299 cell line was used as a control (p53 null) and was purchased from American type culture collection (ATCC) in 2000. Short tandem repeat profiling confirmed authenticity via CellBank Australia (http://www.cellbankaustralia.com/) in 2016. Polyclonal H1299 cell lines (Supplementary Figure S1) expressing **Δ**133p53α or **Δ**33p53β isoforms were established as part of this study, using plasmids expressing **Δ**133p53α or **Δ**133p53β, fluorescently tagged with eGFP at the N terminus (EGFP**Δ**133p53α) or the C terminus (**Δ**133p53βEGFP). A plasmid containing eGFP alone served as the control (p53 control, EGFP; EGFPC1 plasmid, Clontech, Addgene). Plasmids were transfected into H1299 cells using Lipofectamine 3000 Transfection Reagent (Invitrogen, USA), and polyclonal cell lines were established by culturing the cells for at least three passages under neomycin selection. All cells were cultured in a humidified atmosphere containing 5% CO_2_ at 37°C. Cultures were routinely tested for Mycoplasma contamination using the MycoAlert Mycoplasma Detection Kit (Lonza Bioscience, Wellington, New Zealand).

Six murine clonal cell lines stably expressing Δ122p53, previously established, were used with original control cell lines [8, 18]. These included Δ122p53PDAC and ControlPDAC, Δ122p53B16F10 and ControlB16F10, and the murine embryonic fibroblast (MEF) 10.1 (Δ122p53MEF10 and ControlMEF10.1, Supplementary Figure S2). Bone marrow antigen-presenting cells were isolated from wild-type C57BL/6 mice as previously described [20, 21]. On day 5, cells were incubated with lipopolysaccharide (LPS) or conditioned media from Δ122p53B16F10 or ControlB16F10 cells. API5 was immunodepleted using an anti-API5 antibody (ab99307, Abcam, Cambridge, UK), at 10 µg/ml of antibody in conditioned media. TLR4 was inhibited using 30 µM of TAK-242 (Sigma–Aldrich, St. Louis, MO, USA). Viable cells were measured using the trypan blue exclusion assay, a method used to determine the number of viable cells following cell culture. The cells were counted using a Countess 3 FL Cell Counter (Thermo Fisher Scientific, Waltham, MA, USA). The terminal deoxynucleotidyl transferase dUTP nick end labeling assay (TUNEL) assay was used to measure apoptosis using Click-iT Plus TUNEL Colorimetric immunohistochemistry (IHC) Detection (Thermo Fisher Scientific) with methyl green as the counter stain, or the TUNEL Assay Kit—HRP-DAB (Abcam, Cambridge, UK). To determine the percentage of positive cells, the entire cell clot was examined, and the number of positive and total cells was counted.

### 2.3. Enzyme linked immunosorbent assay

Cells were cultured in six-well plates for 72 h in Advanced Dulbecco’s modified eagle medium (DMEM/F12) supplemented with HEPES buffer and GlutaMAX (Thermo Fisher Scientific). The medium was collected, centrifuged, and the supernatant was used for downstream assays. API5 levels in conditioned media were measured using the Human API5 Enzyme linked immunosorbent assay (ELISA) Kit or Mouse API5 ELISA Kit (MyBioSource, San Diego, CA, USA), and IL-6 was measured in conditioned media using the Mouse IL-6 Quantikine ELISA Kit (R and D Systems, Minneapolis, MN, USA) according to the manufacturer’s instructions.

### 2.4. Hematoxylin and eosin staining

Tumors and other tissues were collected, fixed in 10% neutral-buffered formalin for 24 h, processed, embedded, and cut into tissue sections. Hematoxylin and eosin staining was performed using an Expredia Gemini AS Automated Slide Stainer (Thermo Fisher Scientific) at the Histology Otago Micro and Nanoscale Imaging Unit at the University of Otago.

### 2.5. Immunohistochemistry and immunofluorescence

Primary antibodies, antibody dilutions, and antigen retrieval methods are listed in Supplementary Table S1. All IHC and immunofluorescence procedures were automated using the Bond RX system (Leica Biosystems, Wetzlar, Germany), as outlined in the Supplementary Methods.

### 2.6. Flow cytometry

Antibodies used in flow cytometry analyses are listed in Supplementary Table S2. The method, including the gating strategy, followed previously described protocols [10].

### 2.7. Western blotting

The method is outlined in the Supplementary Methods.

### 2.8. Mass spectrometry

Human H1299 stable cell lines underwent processing in two separate repeats (each repeat contained Δ133p53α1, Δ133p53α2, Δ133p53α8, Δ133p53β5, Δ133p53β10, and p53 null cells). Murine cell lines (Δ122p53PDAC, ControlPDAC, Δ122p53B16F10, ControlB16F10, Δ122p53MEF10.1, and ControlMEF10.1) underwent processing in three batches (three technical repeats in each batch). Cell surface proteins were labeled with biotin (0.4 mg, EZ-Link Sulfo-NHS-SS-Biotin, Thermo Fisher Scientific). Streptavidin agarose resin (Thermo Fisher Scientific) was added to the lysed proteins, and the samples were sent to the Centre for Protein Research at the University of Otago for liquid chromatography-tandem mass spectrometry (LC-MS)-based quantitative protein profiling (see Supplementary Methods). Heatmaps were generated using the log_2_-transformed protein abundance ratios. These values were processed in R Studio (version 4.1.1). Δ133p53α and Δ133p53β isoform data were mapped against proteins with increased (abundance ratio > 1.2) or decreased (abundance ratio < 0.8) expression relative to the control. Significance was defined as a consistent increase or decrease in at least two Δ133p53α or two Δ133p53β cell lines across both replicates.

For PDAC, B16, and MEF data, abundance ratios were derived from technical replicates, and proteins were mapped onto heatmaps to those that showed significantly increased and decreased proteins. Significance was defined as fold-change (>1.2 or <0.8) in at least two of three biological replicates.

### 2.9. RNAscope

Formalin-fixed, paraffin-embedded cell clots were cut into 5 µm sections. The RNAscope manual assay used protease plus digestion (20 min) and the 2.5HD red detection kit (Advanced Cell Diagnostics, Newark, CA, USA), following the manufacturer’s instructions. Positive cells were identified using the Aperio Scancope CS digital pathology system (Aperio, Vista, CA, USA). Two blinded examiners evaluated slides. Ten fields (×400 magnification) were chosen, and the percentage of positive cells per total cells was counted. If no positive cells were observed in 10 random fields, the entire tissue section was screened. Probes included murine ubiquitin C (*UBC*; positive control), *DapB* (negative control), and *IL-6* (advanced cell diagnostics).

### 2.10. Statistical analyses

Results are reported as mean ± SD. Statistical analyses of continuous variables were performed using Student’s *t-*test or one-way ANOVA corrected for multiple comparisons. Categorical variables were analysed using Fisher’s exact test. Survival and tumor progression analyses were performed using the log-rank test (Mantel–Cox test). Survival analyses for the animal study and the comparison of metastases among the treatment groups were corrected for multiple comparisons using the Bonferroni correction method. Analyses were performed using Prism 10 software (GraphPad, San Diego, CA, USA).

## 3. Results

### 3.1. Inhibiting cell surface trafficking reduces tumor growth in Δ122p53 tumors

**Figure 1:**
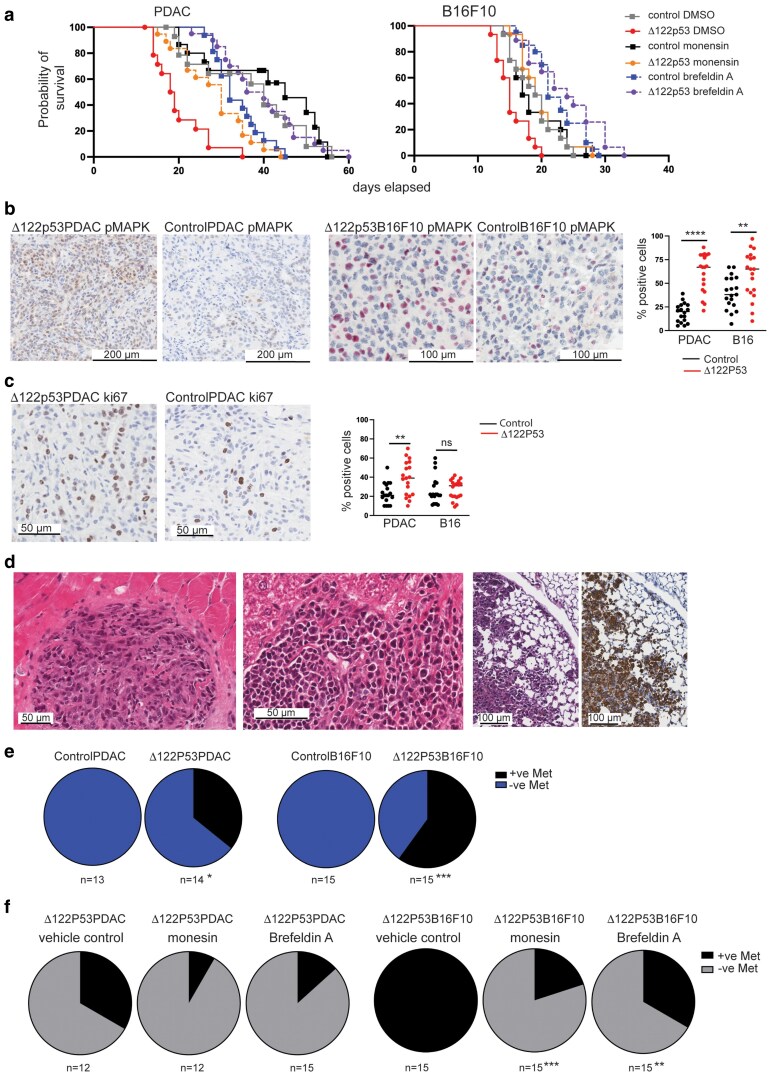
Inhibiting changes to the cell surface altered properties of Δ122p53 expressing tumors. (a) Survival of C57BL/6 mice syngrafted with Δ122p53 expressing, or control PDAC and B16F10 melanoma cells and treated with cell surface signaling inhibitors (monensin and brefeldin A). (b) Immunohistochemistry staining of Δ122p53 and control subcutaneous tumors for pMAPK as a measure of MAPK pathway activation. Representative images (left) and quantification of immunohistochemistry staining (right). (c) Immunohistochemistry staining of Δ122p53 and control subcutaneous tumors for ki67 as a measure of proliferation. Representative images (left) and quantification of immunohistochemistry staining (right). (d) Local invasion and metastasis of Δ122p53 expressing tumors. From left, a Δ122p53PDAC tumor in skeletal muscle, a Δ122p53B16F10 tumor metastasis to the liver, and a Δ122p53B16F10 tumor metastasis to the lung. (e) Δ122p53 expressing tumors had an increased incidence of metastasis compared with control tumors. (f) Δ122p53 expressing tumors treated with monensin and brefeldin A had reduced incidence of metastasis in a second cohort of animals treated for a continuous 10-day period. Results mean ± SD. *P < .05, **P < .01, ***P < .001, and ****P < .0001, comparisons relative to the control. Met, metastasis; −ve, negative; +ve, positive.

To elucidate the importance of changes at the Δ133p53 cell surface in tumor progression, cell surface trafficking was inhibited in mice syngrafted with Δ122p53-expressing cell lines. All mice syngrafted with PDAC and B16F10 cells developed tumors. All Δ122p53-expressing PDAC and melanomas grew faster than control tumors (Fig. 1a), and, consistent with this, all Δ122p53-expressing tumors showed increased markers of proliferation with more phosphorylated mitogen-activated protein kinase (pMAPK) and ki67-positive cells compared with control cell lines (Fig. 1b and c, respectively).

**Figure 2.**
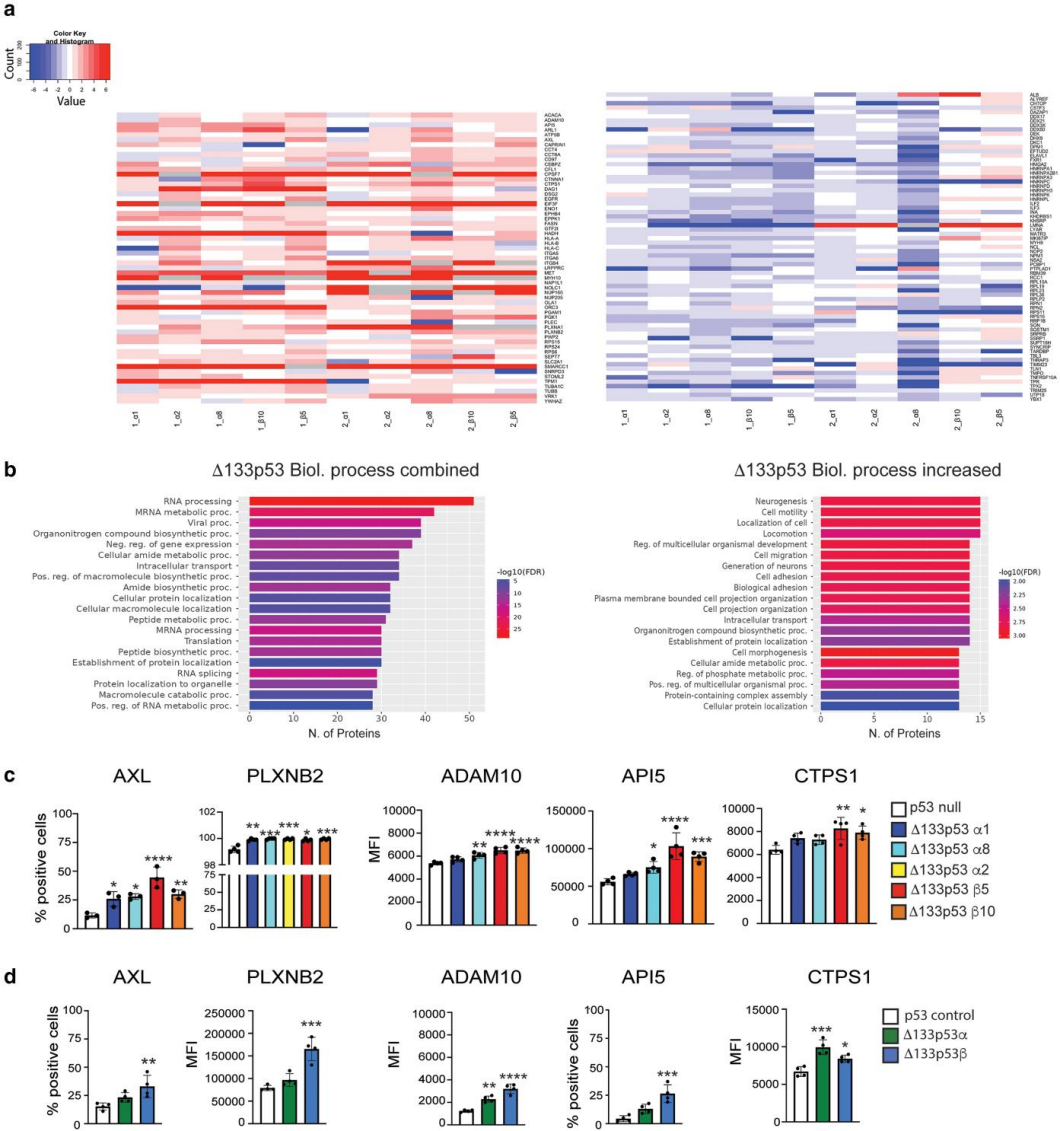
Proteins altered on the cell surface of Δ133p53 expressing cells. (a) Heatmaps showing proteins increased (left) or decreased (right) on the Δ133p53 cell surface from mass spectrometry measurement of biotinylated cell surface proteins. Log2-transformed protein abundance ratios of Δ133p53 (α or β)/H1299 (control) proteins. Abbreviations: Experiment 1 data: 1α1 = Δ133p53α1, 1α2 = Δ133p53α2, 1α8 = Δ133p53α8, 1β5 = Δ133p53β5, 1β10 = Δ133p53β10. Experiment 2 data: 2α1 = Δ133p53α1, 2α2 = Δ133p53α2, 2α8 = Δ133p53α8, 2β5 = Δ133p53β5, and 2β10 = Δ133p53β10. Graphics produced using ShinyGO 0.76 [22]. (b) Gene ontology analyses of proteins altered (increased and decreased proteins combined (left) and increased only (right)) on Δ133p53α and Δ133p53β H1299 clonal cell surface. (c) Flow-cytometry analysis to confirm proteins increased on H1299 clonal cell lines expressing Δ133p53α, and Δ133p53β. (d) Flow-cytometry analysis to confirm proteins increased on H1299 polyclonal cell lines expressing Δ133p53α, and Δ133p53β. Results mean ± SD. *P < .05, **P < .01, ***P < .001, and ****P < .0001. MFI, mean fluorescence intensity.

Inhibiting cell surface trafficking using monensin and brefeldin A was associated with reduced tumor growth for Δ122p53PDAC and Δ122p53BF10 tumors (Fig. 1a). Mice with Δ122p53PDAC tumors showed increased median survival from 18.5 days with the vehicle control to 30 days with monensin (*P* = .0066, hazard ratio = 2.55, 95% CI 1.13–5.79) and to 38 days with brefeldin A treatment (*P* < .0001, hazard ratio = 4.76, 95% CI 1.74–13). Brefeldin A was more effective at reducing tumor growth compared with monensin in Δ122p53PDAC tumors (*P* = .0015, hazard ratio = 2.69, 95% CI 1.29–5.6). No difference in tumor growth was observed with the administration of monensin or brefeldin A in the ControlPDAC tumors. Mice with Δ122p53B16F10 tumors showed increased median survival from 15 days with the vehicle control to 19 days with monensin (*P* = .0009, hazard ratio = 2.95, 95% CI 1.3–6.71) and to 24 days for brefeldin A treatment (*P* < .0001, hazard ratio = 3.81, 95% CI 1.58–9.24). Brefeldin A was again more effective at reducing tumor growth compared with monensin (*P* = .0432, hazard ratio = 2.16, 95% CI 1–4.65) for Δ122p53-expressing tumors. For animals with control tumors, no difference in tumor growth was observed with the administration of monensin, but brefeldin A did improve survival for the ControlB16F10 tumors (median survival 19 versus 21 days; vector vehicle control versus brefeldin A treatment (*P* = .021, hazard ratio = 2.22, 95% CI 1.04–4.76).

In addition to enhanced tumor growth, histological examination of tissues showed evidence of metastasis in mice with Δ122p53-expressing, but not control, tumors (Fig. 1d). Δ122p53PDAC metastasized in 36%, and Δ122p53B1610 tumors metastasized in 60% of mice to at least one organ (Fig. 1e). In cohorts reaching the tumor size endpoint, no difference in the incidence of metastasis was observed between Δ122p53PDAC and Δ122p53B16F10 tumors in animals treated with brefeldin A or monensin. However, because the treatment period was not continuous and some animals survived beyond the treatment period, a 10-day continuous treatment group was used to investigate whether cell surface inhibitor treatment could reduce metastasis. Metastases were significantly fewer in animals with Δ122p53B16F10 tumors following treatment with monensin and brefeldin A (Fig. 1f, *P* = .003 and *P* = .0248 for monensin- and brefeldin A-treated tumors, respectively), but neither treatment reached significance for reducing the incidence of metastasis in mice engrafted with the Δ122p53PDAC cell line. The findings from inhibiting cell surface trafficking revealed that changes at the cell surface were important for the added tumor-promoting properties in Δ122p53-expressing tumors.

### 3.2. Characterization of proteins altered on the Δ133p53/Δ122p53 cell surface

To characterize the proteins altered at the Δ133p53 cell surface, cell surface proteins on Δ133p53 and Δ122p53 cells were identified by mass spectrometry following labeling of cell surface protein with biotin. In Δ133p53α and/or Δ133p53β clonal cell lines, 61 proteins were increased and 72 proteins decreased (Table 1 and Fig. 2a). Five proteins (EGFR, ENO1, ITGA5, c-MET and SLC2A1) have been identified in other studies [10, 12–14]. Increased levels of other proteins play well-characterized roles in the inhibition of apoptosis (API5, ADAM10, and YWHAZ) and promotion of metastasis (AXL, CD97, EPHB4, ITGA6, PLXNB2, STOML2, TUBA1C, and TUBB). Other proteins included ARL1, which is involved in membrane trafficking, the major histocompatibility complex class I (HLA A-C), and proteins involved in RNA or ribosome function (PWP2, CAPRIN1, CPSF7, CTPS1, SNRPD3, RSPS15, RPS24, and RSP6). The strong representation of cancer-promoting, RNA/ribosome-related proteins was evident when using Gene Ontology with the list of increased and decreased proteins from Δ133p53α and β cell lines combined (Fig. 2b). The pathways associated with increased protein levels included cancer-related pathways, cell motility, and cell migration. Increased AXL, PLXNB2, ADAM10, API5, and CTPS1 on the Δ133p53 cell surface was confirmed using flow cytometry (Fig. 2c and d).

**Table 1.** Proteins altered on the Δ133p53 cell surface following mass spectrometry measurement of biotinylated labeled cell surface proteins.

Proteins increased			Proteins decreased		
Δ133p53α and Δ133p53β cells	Δ133p53α cells	Δ133p53β cells	Δ133p53α and Δ133p53β cells	Δ133p53α cells	Δ133p53β cells
ACACA; ATP5B; AXL^a^; CTPS1^a,b^; DSG2; FASN; GTF2I; H3F3-(A,AP4,B)^b^; ITGA6; MET^a,b^; PLXNB2^a^	ARL1; CAPRIN1; CEBPZ; DAG1; EGFR^a^; EIF3F^b,c^; ENO1^a^; EPHB4; EPPK1; HADH; HIST1H3-(A-J)^b^; HLA-(A-C); ITGB4; MYH10^b,c^; NAP1L1; NUP-(160^b^, 205); OLA1; ORC3; PLEC; PLXNA1; RSP-(S15, 6); SLC2A1^a^; SMARCC1^b,c,d^; SNRPD3^d^; STOML2; TUBA1C; TUBB; VRK1	ADAM10^a^; API5^a,d^; CCT-(4,6A;) CD97; CFL1; CPSF7^b,c^; CTNNA1^d^; ITGA5; LRPPRC; NOLC1; PGAM1; PGK1; PWP2; RPS24; SEPT7; TPM1^b^; YWHAZ	NPM1; PCBP1; SUP16H; TARDBP; UTP18	ALB; ALYREF; CHTOP^b^; DAZAP1^b^; DDX-(17, 21, 3X); DEK; DHX9; DKC1; DPM1; ELAVL1^b^; FXR1; HMGA2; HNRNP-(A1^b^, A2B1^b^, A3, C, D, H3, K, L^b^); ILF (2, 3); INA; KHDRBS1^b^; KHSRP^b^; LMNA; LYAR; MKI67IP; MYH9; NCL^b^; NOP2; NSA2; PTPLAD1^b^; RBM39; RCC1; RPL-(19, P2); RPN2; RPS16; RRP1B^b^; SERPINH1; SON; SQSTM1; SRPRB; SSRP1; SYNCRIP; TBL3, TIMM23^b^, TLN1, TMPO; TNFRSF10A; TPR^b^; TPX2^b^; TRIM25; YBX1	CSTF3; DDX50^b^; EFTUD2; KIRREL; MATR3; RPL10A; RPL-(23,36); RPN1; RPS11

Proteins measured in H1299 clonal cell lines expressing Δ133p53α and Δ133p53β compared with the control (parental H1299 cells). Abundance ratios (increased (>1.2-fold) or decreased (<0.8-fold) in at least two Δ133p53α and two Δ133p53β cell lines across both repeats compared with the parental H1299 cells).

^a^Proteins increased confirmed by flow cytometry or other studies.

^b^Proteins with greater changes in abundance ratios (>2-fold or <0.5-fold).

^c^Only found on the Δ133p53 cell surface.

^d^Proteins also altered similarly in Δ122p53 analyses.

Proteins increased on the Δ122p53 cell surface (Table 2) were compared with those observed on the Δ133p53 cell surface to identify commonly altered proteins. API5 expression was increased across all Δ133p53 and Δ122p53 cell lines, and four other proteins (CTNNA1, EPPK1, SNRPD3, and SMARCC1) were increased in at least one human and one murine cell line. No similarities were observed in protein levels between individual human and murine cell lines. As API5 was increased across all Δ133p53 and Δ122p53 cell lines, we next investigated the consequences of increased API5 on the Δ133p53/Δ122p53 cell surface.

**Table 2.** Proteins altered on the Δ122p53 cell surface following mass spectrometry measurement of biotinylated labeled cell surface proteins.

Proteins increased on all three Δ122p53 murine cell lines	Proteins increased on two of three Δ122p53 murine cell lines	Proteins decreased on all three Δ122p53 murine cell lines	Proteins decreased on two of three Δ 122p53 murine cell lines
API5; BAZ1B; CSNK2A1; DRG1; HADHA; HNRNPC; PARP; PP1A; SF3B1; SMC3; SQSTM1; TOP1	APEX1; BANF1; BOP1; CTNNA1; DDOST; DDX-(21, 39B, 3X, 47, 5, 50); EEF-(1A1, 2, 2S1); EIF-(2S3X, 3B, 4A3); ELAVL1; EPPK1; EZR; FBL; FGB; FLNC; FN1; HNRNP-(A1, A3, AB, F, K, L, LL, M, U); HSP90B1; IGF2BP1; FTSJ3; GLYR1; H1-(2, 3); HBA-A1; HDAC2; HNRNPA0; ILF2; IMP3; KHDRBS1; KRT1; KRT78; KTN1; LMNB1; MACROH2A1; MATR3; MCG-(134299, 18249, 19351, 49954); MCM5; MRTO4; MTA2; NCL; NHP2; NIFK; NOL6; NOP-(56, 58;) NPM3; NUP155; PCBP1; PCBP2; PFN1; PGK1; PHGDH; PPP1CC; PT-(BP1, BP3); RAD21; RBM-(19, X); RCL1; RNPS1; RPF2; RPL-(12, 17, 27A, 6); RPN1; RPS23; RRS1; RTCB; SAP18; SERBP1; SFPQ; SGPL1; SLC25-(A4, A5); SLTM; SMARC-(A5, C1); SMC2; SMU1; SND1; SNRP-(A1, D3); SSRP1; SUPT16; THRAP3; TMPO; TOP2-(A, B); UTP-(14A, 15); VIM; WDR82	ITGB1 KRT19; KRT42	AHNAK ACTB ALDH2 ATP5C1 ANX-(A1, A3) ATP-(1A1, 5C1) CD44 CDH1 COX5A CTNNB1 GAPDH; HADH mCG-(10343, 145246, 18900) NEDD4 PDIA3 PRDX6 SPTBN1 UQCRC2

Proteins measured on Δ122p53PDAC, Δ122p53B16F10, or Δ122p53MEF cell lines compared with the corresponding control cell line ControlPDAC, ControlB16F10, and ControlMEF, respectively. Abundance ratios (increased (>1.2-fold) or decreased (<0.8-fold) in Δ122p53 cells compared with corresponding control cells within a technical repeat).

### 3.3. API5 increases TLR4 signaling in Δ133p53/Δ122p53 cells

**Figure 3.**
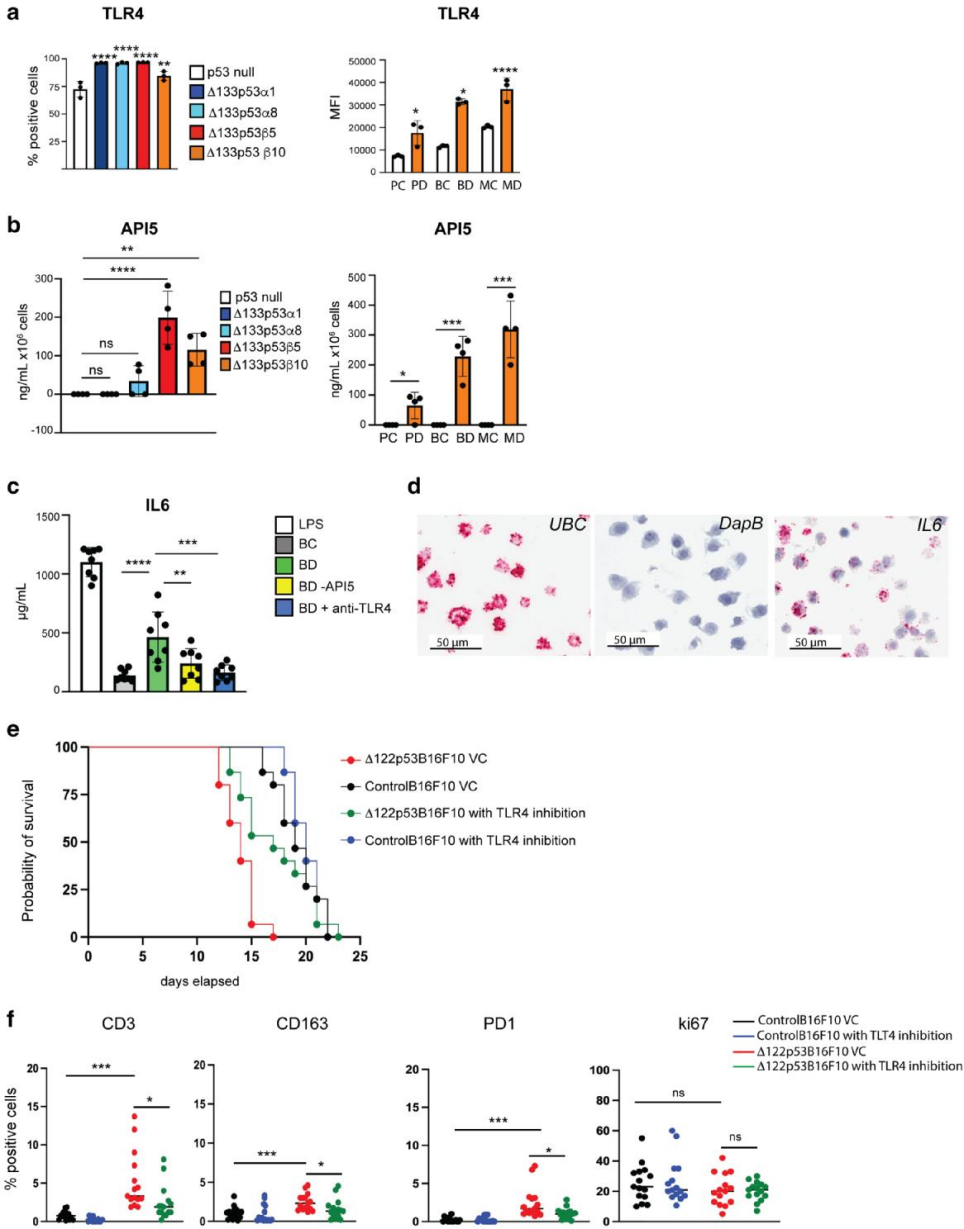
Δ133p53 and Δ122p53 expression was associated with increased TLR4 function. (a) Flow-cytometry analysis to confirm increased TLR4 on the cell surface of Δ133p53α, and Δ133p53β (left) and Δ122p53 (right) expressing cells. (b) Measurement of the TLR4 agonist, API5, by ELISA in cell media from Δ133p53 (left) and Δ122p53 (right) expressing cell lines. (c) Measurement of the downstream target of TLR4 signaling, IL6, by ELISA in cell media from bone marrow derived antigen-presenting cells following incubation with conditioned media from: B16F10 (BC) control or B16F10Δ122p53 cells (BD), B16F10Δ122p53 cells with API5 immunodepleted (BD-API5), and B16F10Δ122p53 cells incubated with the TLR4 inhibitor (TAK-242, +anti-TLR4). (d) Confirmation that *IL6* was produced in bone marrow derived antigen-presenting cells following incubation with conditioned media from B16F10Δ122p53 cells using RNAscope on cell clot sections. *UBC*, ubiquitin C (positive control); *DapB*, (negative control). (e) Survival of C57BL/6 mice syngrafted subcutaneous with Δ122p53 expressing or control B16F10 cells following treatment with the TLR4 inhibitor (TAK-242) or the vehicle control (DMSO). (f) Reduction of immune infiltration in Δ122p53 expressing tumors following TLR4 inhibition. Quantification of CD3, PD1, CD163, and ki67-positive cells using immunohistochemistry in subcutaneous tumors. Murine cell lines: PC, pancreatic ductal adenocarcinoma (PDAC) cell line control; PD, PDAC cell line expressing Δ122p53; BC, B16F10 melanoma control cell line; BD, B16F10 melanoma expressing Δ122p53 cell line; MC, murine embryonic fibroblast (MEF) 10.1 control cell line; MD, MEF10.1 cells expressing Δ122p53. Comparisons between Δ122p53 expressing cells with and without TLR4 inhibition and between Δ122p53 expressing and control tumors without TLR4 inhibition. Results mean ± SD. *P < .05, **P < .01, ***P < .001, and ****P < .0001.

Given that API5 was increased on the Δ133p53/Δ122p53 cell surface and its potential as a TLR4 agonist, we first investigated whether TLR4 was increased. Using flow cytometry, TLR4 was observed to be increased on the cell surface in all Δ133p53 and Δ122p53 expressing cells (Fig. 3a). To determine the potential for increased TLR4 stimulation, we investigated whether API5, which increased at the cell surface of Δ133p53 and Δ122p53, was secreted and functioned as a TLR4 agonist. API5 levels were increased in conditioned media from Δ133p53β and Δ122p53 and in some Δ133p53α-expressing cells compared with the control (Fig. 3b). The API5 released could activate TLR4 signaling, as evident with increased IL-6 produced from bone marrow-derived antigen-presenting cells from wild-type C57BL/6 mice incubated with conditioned media from Δ122p53B16F10 cells (Fig. 3c). The amount of IL-6 produced was reduced with conditioned media that was immunodepleted of API5 and included the TLR4 inhibitor (TAK-242, Fig. 3c). To confirm that IL-6 detected originated from antigen-presenting cells rather than Δ122p53B16F10 cells, IL-6 mRNA expression were confirmed using RNAscope (Fig. 3d). These findings showed that increased API5 produced from Δ133p53/Δ122p53 cells could act as a TLR4 agonist.

### 3.4. Inhibiting TLR4 signaling reduces Δ122p53 tumor growth

Our findings suggested that TLR4 stimulation is increased in Δ133p53 and Δ122p53 cells. To determine whether increased TLR4 function was responsible for the increased tumor growth and metastasis with Δ122p53, tumor cell lines were again syngrafted into recipient mice, followed by treatment with the TLR4 inhibitor TAK-242. Inhibition of TLR4 increased the survival of mice engrafted with Δ122p53B16F10 tumors (median survival 17 versus 14 days, *P* = .0018, hazard ratio = 2.4, 95% CI 1.11–5.52; Δ122p53B16F10 treated with the TLR4 inhibitor versus Δ122p53B16F10 vehicle control, Fig. 3e). No difference with TLR4 inhibition was observed for animals engrafted with ControlB16F10 cell lines (median survival 20 versus 19 days, *P* = .4414, hazard ratio = 1.2, 95% CI 0.61–2.56; ControlB16F10 tumors treated with the TLR4 inhibitor versus ControlB16F10 vehicle control, Fig. 3e). Analysis of CD3, CD163, PD1, and ki67 in syngrafted tumors showed that TLR4 inhibition was associated with reduced CD3, PD1, and CD163 infiltration in Δ122p53B16F10 tumors (Fig. 3f). ControlB16F10 tumors showed no difference in ki67, CD3, PD1, or CD163 expression upon TLR4 inhibition (Fig. 3f).

These findings showed that inhibiting TLR4 signaling could reduce tumor growth and immunosuppressive cell infiltration in Δ122p53 tumors.

## 4. Discussion

Here, we report that changes at the cell surface are responsible for the increased tumor growth and metastatic effects associated with Δ133p53/Δ122p53 function. Two prominent changes at the cell surface that can contribute to the tumor microenvironment through auto/paracrine stimulation include increased TLR4 and increased secretion of the TLR4 agonist API5. These results highlight the role of Δ133p53/Δ122p53 in directly influencing the tumor microenvironment and provide much-needed therapeutic possibilities for targeting Δ133p53.

Given that multiple cancer-promoting proteins are altered on the surface of Δ133p53 cells, multiple choices are available for selecting drug targets. Here, inhibition of TLR4 was chosen as the strategy to target Δ122p53 tumors, highlighting that targeting a specific protein can reduce the tumor-promoting properties associated with Δ122p53. The increased TLR4 on Δ133p53 cells suggests that Δ133p53-expressing cells may be more predisposed to TLR4 agonists in the microenvironment. Potential TLR4 agonists could include apoE and LPS based on other studies [23, 24]. Several studies have proposed a role for TLR4 in cancer progression and resistance [25–27]. TLR4 increases inflammatory responses and tumor growth following paclitaxel, suggesting TLR4 may be the underlying cause of chemotherapy resistance [28]. This evidence suggests that inhibiting TLR4 could be an effective treatment strategy against cancer. However, not all studies have proposed inhibiting TLR4 as a cancer treatment; other studies have proposed an alternative strategy involving its stimulation, which has been associated with reduced tumor growth and better prognosis [29, 30]. Furthermore, a lower serum TLR4 concentration has been reported in patients with metastatic tumors than in those with nonmetastatic tumors [29, 30].

The difficulty in determining whether TLR4 should be inhibited or stimulated during cancer treatment is further exacerbated by the different effects of TLR4 on the immune system. The role of TLR4 in recruiting TAMs into the tumor microenvironment, as evidenced in TLR4-null mice and the correlation between TLR4 and TAMs in human cancers [31, 32], supports the role of TLR4 in cancer treatment. However, in the current study, TLR4 inhibition led to reduced T cell infiltration. In addition, other studies have reported that TLR4 agonists stimulate PD-L1, suggesting that TLR4 agonists combined with immune checkpoint inhibitors could enhance immunotherapies [33, 34]. The increased expression of TLR4 and API5 in Δ133p53-expressing cells has implications for other conditions. Excessive activation of TLR4 is thought to contribute to age-related conditions [35] and recombinant API5 protects Paneth cells from death by restoring Paneth cell numbers and preventing intestinal injury due to inflammatory insults [36]. Given that Δ133p53 tumors have increased TAMs, PDL-1, and T cells, investigation of inhibition or stimulation of TLR4 is warranted as a potential therapeutic strategy for cancer and potentially extended to other conditions [5, 6, 10].

The mechanism by which Δ133p53 increases proteins on the cell surface is uncertain and was not investigated in this study. As many of the classic cancer-promoting proteins that increase on the Δ133p53 cell surface have been observed to increase on the cell surface of cells with mutant p53 [15, 16, 37], it could be that Δ133p53 functions similarly to mutant p53. Mutant p53, which is associated with increased cancer-promoting proteins on the cell surface, may be involved in Rab coupling protein-mediated trafficking [16, 37–39]. The results of this study highlight the role of other proteins on the cell surface, many of which are not traditional cell surface proteins. Most of the proteins altered on the Δ133p53 and Δ122p53 cell surface were related to RNA processing and the ribosome. However, it remains unclear why these proteins are altered on the cell surface. Recently, ribonucleoproteins have been identified as part of the focal adhesion machinery in cancer cells, strengthening B cell responses [40]. Whether the alteration of ribonucleoproteins on the Δ133p53 cell surface is another means by which Δ133p53 affects immune responses requires further experimentation.

Limitations of this study include the use of overexpressing cell lines; therefore, whether the same effects on the cell surface could be achieved with endogenous Δ133p53 expression is uncertain. However, the amount of Δ133p53β found in overexpressing cell lines was comparable with that found in brain metastases [10, 11]. This study investigated Δ133p53 and Δ122p53 isoforms largely in the absence of the complexity of other p53 moieties that could modulate the effects of individual Δ133p53 isoforms on how proteins are expressed on the cell surface. The effects of different p53 proteins are an important consideration, given that inhibiting TLR4 is a suitable treatment strategy for mutant p53 cancer but not for tumors with wild-type p53 function [41].

In summary, we report that Δ133p53 isoforms are associated with alterations at the cell surface, suggesting numerous opportunities for targeting Δ133p53-expressing tumors, including the inhibition of TLR4. Some of the changes at the Δ133p53 cell surface affect both the ligand and the corresponding receptor, highlighting that inhibition of cell surface signaling pathways may restrain various effects of Δ133p53.

## Supplementary Material

bgaf051_Supplementary_Data

## Data Availability

Mass spectrometry data sets are available as Supplementary File S3 and heatmaps (Supplementary File S4). Example R studio code for creating heatmaps of proteins increased and decreased from the mass spectrometry data is given in Supplementary File S5.
