# Supplementary material for: Δ133p53 isoform enhances TLR4 function to promote tumor growth
Source: Carcinogenesis. 2025 Aug 29;46(4):bgaf051. doi: 10.1093/carcin/bgaf051 (PMC12683340; doi:10.1093/carcin/bgaf051)
Supplement: bgaf051_Supplementary_Data [file bgaf051_supplementary_data.zip › Supplementary Material Combined.docx]

**Supplementary Material and Methods, Supplementary Figure Legends and Description of Supplementary Files, and Supplementary Tables**

**Immunohistochemistry and immunofluorescence**

Cells were harvested and made into cell clots using Expredia HistoGel Specimen Processing Gel (Thermo Fisher Scientific, Waltham, MA, USA). Cell clots were fixed in 10% neutral buffered formalin (NBF), processed and embedded in paraffin wax. H1299 polyclonal cell lines were also grown on poly-L-lysine (Sigma-Aldrich, St. Louis, MO, USA) coated 13MM coverslips (Paul Marienfeld GmbH &Co. KG, Lauda-Königshofen, Germany). Following cell culture coverslips were fixed in 4% paraformaldehyde and used in immunofluorescence based assays.

For cell clot preparations and murine tissues, 4 µm sections from formalin fixed paraffin-embedded tissues were used. Immunohistochemistry analysis on human tissues used the BOND Polymer Refine detection kit (Leica Biosystems) according to the manufacturer instruction’s. Immunohistochemistry analyses on murine cell clots and tissues used the BOND Polymer Refine detection kit (PDAC and MEF10.1 cells) or BOND Polymer Refine RED Detection kit (B16F10 cells, Leica Biosystems) with modification where the mouse and rabbit dual polymer was replaced with a rabbit only polymer (EDL (Dako, Agilent, Technologies, Santa Clara, CA, USA) for DAB chromogen, and MACH 2 Rabbit-AP-Polymer (Biocare Medical, Pacheco, CA) for Fast Red chromogen. Immunofluorescence used Alexa Fluor 594 secondary antibodies (Thermofisher Scientific). Antibodies were diluted in Primary Antibody Diluent BOND (Leica Biosystems, Wetzlar, Germany) or Antibody Diluent, Background Reducing (Agilent Dako, Santa Clara, CA, USA). For chromogenic-based staining, slides were mounted in DPX Mountant (Sigma-Aldrich) and positive cells were identified using the Aperio Scancope CS digital pathology system (Leica Biosystems). For fluorescent staining, slides were counterstained using DAPI mounted on glass slides with Fluoromount-G Mounting Medium (Thermofisher Scientific). Slides were evaluated using the Aperio VERSA digital pathology system (Leica Biosystems). Slides were evaluated by two blinded examiners. For determining the percentage of positive cells ten high powered fields (x400 magnification) were selected at random and the number of positive cells and total cells counted.

For analyses where cells were grown on coverslips EGFPΔ133p53α and Δ133p53βEGFP expressing H1299 cells were stained with primary antibodies (Supplementary Table 1) and the secondary Alexa Fluor 594 or Alexa Fluor 488 goat anti-rabbit antibodies (Thermo Fisher Scientific). Cells were visualised using the Lionheart FX automated microscope (BioTek, Winooski, VT, USA) or the Aperio VERSA digital pathology system (Leica Biosystems). For analyses using the Aperio VERSA digital pathology system ten high power field (x400 magnification) snapshots were taken, and positive and total number of cells were counted to determine the percentage of positive cells. Slides were evaluated by two blinded examiners.

**Western blotting**

Following cell culture, cell pellets were lysed using radioimmunoprecipitation assay buffer (RIPA) with protease/phosphatase inhibitor added (Pierce Protease and Phosphatase Inhibitor, Thermo Fisher Scientific). The BCA assay was used to measure total protein concentration (Pierce BCA Protein Assay Kit). Cell lysates (50-100 μg) were headed in SDS sample buffer, separated on SDS-polyacrylamide gels and then electroblotted onto a nitrocellulose membrane (0.2 µm, Amersham Protran Premium, Merck, Auckland, New Zealand). Membranes were incubated with primary antibodies at room temperature overnight (Supplementary Table 1). Secondary antibodies IRDye, dilution: 1/15000, Licorbio, Lincoln, NE, USA) were added for 1 hour at room temperature. Immunoreactive protein bands were detected using the Odyssey Scanning System (Licorbio).  Loading controls (Supplementary Figure 1) were used to control for protein loading. To quantify the protein concentration detected on the nitrocellulose membrane, densitometry analysis was performed using Image Studio Lite software (Licorbio). The protein of interest was normalised against a loading control. Protein expression levels were then plotted as mean fluorescence intensity (MFI).

**Mass spectrometry**

Biotin 0.4mg (EZ-Link Sulfo-NHS-SS-Biotin, Thermo Fisher Scientific) was added to each well, six wells total, with each well having at least 250,000 cells. Plates were kept in the dark on ice for 30 minutes. Sample aliquots were investigated using western blotting to ensure biotin labelling was successful. Next, each well was washed with ice-cold DPBS six times and lysis buffer (25mM HEPES+1% (v/v) Triton X-100+10% (v/v) glycerol at pH 8.2 with protease/phosphatase inhibitor cocktail) added (Thermo Fisher Scientific). Streptavidin agarose resin (Thermo Fisher Scientific) was added to lysed proteins and incubated at 4^0^C overnight. Samples washed five times in biotin wash buffer (50mM EDTA, 10mM Tris pH 7.4, 1% NP-40, 0.4% Sodium deoxycholate) and samples sent to the Centre for Protein Research (CPR) at the University of Otago for liquid chromatography coupled mass spectrometry (LCMS) based quantitative protein profiling.

On bead samples were briefly washed with 100 mM Triethylammonium bicarbonate (TEAB) buffer and were subjected to nucleic acid lysis using Denarase enzyme (c-LEcta, Leipzig, Germany). The on bead proteins were digested with trypsin following the reduction and alkylation of cysteines. The resulting tryptic peptides were analyzed using the UltiMate 3000 Nano-flow uHPLC-System (Dionex, CA, USA) coupled to the LTQ Orbitrap XL Mass Spectrometer (Thermo Fisher Scientific). Peptides were separated on an in-house packed 20 cm emitter-tip column (75 um ID PicoTip fused silca tubing, CoAnn Technologies, Richland, WA, USA) packed with Aeris C-18, 2.6 µM material (Phenomenex, Torrance, CA, USA). Peptides were eluted from the column using an increasing acetonitrile (ACN) gradient (from 5% [v/v] ACN, 0.1% [v/v] formic acid to 99% [v/v] ACN, 0.1% [v/v] formic acid in water) at a flow rate of 300 nl/min for 2 hours (the gradient consisted of: 5 to 25% ACN in 78 mins, 25 to 40% ACN in 10 mins and 40 to 99% ACN in 3 mins). The eluted peptides were sprayed into the LTQ Orbitrap XL Mass Spectrometer,. The strongest 11 MS1 precursors between 400-2000 m/z measured in the orbitrap mass analyser were selected for collision induced dissociation (CID) to generate MS2 or MS/MS spectra in the linear trap quadrupole (LTQ) ion trap. Dynamic exclusion was enabled with 2 repeat counts during 30s and an exclusion period of 180s.

For protein identification mass spectrometry data was queried against the NCBI Human or Mouse sequence database (downloaded in March 2019) with the SequestHT search engine node within the Proteome Discoverer software (version 2.4) (Thermo Fisher Scientific). Search parameters were configured to identify tryptic peptides with a maximum of 2 missed cleavage sites. Variable modifications included oxidised methionine and deamidation of asparagines and glutamines, while carbamidomethyl cysteine was treated as a static modification. The precursor mass tolerance threshold was 10 ppm and the maximum MS2 fragment mass error was 0.5 Da. In the quantitative analysis, data normalisation was performed based on the sum of abundances from all detected peptides across all samples. The calculation of relative protein abundance adopted the Top-3 approach, averaging the three most abundant peptides for each specific protein(1).

To identify proteins increased or decreased on the Δ133p53 or Δ122p53 cell surface abundance ratios (Δ133p53 cell line/p53 control for each replicate, and median abundance ratio across the three technical repeat for an individual ‘biological’ repeat for Δ122p53 expressing cell line/median abundance ratio across the three technical repeat for an individual ‘biological’ repeat for the corresponding control cell line) >1.2 for increased proteins, or <0.8 for decreased cell lines were selected. A further filtering process was used to select the final set of increased and decreased proteins. For human proteins the final list of proteins were those increased or decreased in at least two Δ133p53α and/or two Δ133p53β cell lines in one repeat and with at least two of the same cell lines showing increased or decreased proteins in the second repeat. For murine cell lines the final list of proteins were those with identified as increased or decreased in at least two biological repeats.

Abundance ratios were formatted into a matrix using R packages dplyr_1.1.4(1) and tidyr_1.3.0(2) allowing generation of heatmaps using heatmap.2 the ‘enhanced heatmap’ function of gplots_3.1.3.1(3) and colour gradients from RColorBrewer_1.1-3(4).

**Supplementary Figure 1.** Characteristics of murine cell lines expressing Δ122p53. (**A**) Confirmation that Δ122p53 cells expressed the Δ122p53 protein using western blotting (left). Loading control, α tubulin. Quantification of p53 relative to the loading control (right). Murine cell lines: PC, pancreatic ductal adenocarcinoma (PDAC) cell line control; PD, PDAC cell line expressing Δ122p53; BC, B16F10 melanoma control cell line; BD, B16F10 melanoma expressing Δ122p53 cell line; MC, murine embryonic fibroblast (MEF) 10.1 control cell line; MD, MEF 10.1 cells expressing Δ122p53. +ve C (positive control, Δ133p53α8 H1299 clonal cell line). (**B**) Trypan blue exclusion assay for ascertaining live cells. Results are mean ± SD. ** P < 0.01. (**C**) TUNEL assay to identify apoptotic cells. +ve, control, positive control using DNase I. (**D-E**) Trypan blue exclusion assay for estimation of cell viability with incubation of different concentrations of cell surface trafficking compounds (monensin (**D**) or brefeldin A (**E**)). Cell surface trafficking inhibitors were incubated with PDAC and B16F10 cell lines for 6 or 24 hours before measurement. No statistically significant differences were found. Results were normalized to the number of cells at 0 hours.

**Supplementary Figure 2.** Characteristics of polyclonal H1299 cells expressing Δ133p53α or Δ133p53β. (**A**) Confirmation that polyclonal cells H1299 expressed Δ133p53α (EGFPΔ133p53α) and Δ133p53β (Δ133p53βEGFP) using western blotting; loading control (histone 3, green). Lane 1, EGFP only (p53 control); Lane 2, Δ133p53α8 as a positive control for Δ133p53α; Lane 2, Δ133p53β5 as a positive control for Δ133p53β; Lane 3, EGFP Δ133p53α; Lane 5, Δ133p53βEGFP. A non-specific band similar in size to Δ133p53 α and a much lower molecular weight band were also present. (**B**) Quantification of p53 isoforms from western blots, with fluorescence normalized to the loading control. (**C**) Trypan blue exclusion assay for ascertaining live cells. (**D**) TUNEL assay for apoptosis to illustrate the lack of apoptotic cells in all cell lines. +ve, positive control DNase I treated. (**E**) Representative images of immunohistochemical staining for ki67 (left) and quantification of positive cells across cell lines (right). (**F**) Representative images of immunofluorescent staining for nuclear factor kappa B (NF-kB)/p65 staining (left) and quantification of positive cells across cell lines (right). (**G**) Representative images of immunofluorescent staining for pMAPK and EGFP. Scale bar 200 µm. (**H**) Representative images of immunofluorescent staining for PDL1 and EGFP. Scale bar 200 µm. Immunofluorescence and immunohistochemistry analyses performed on cell clots expressing EGFP Δ133p53α, Δ133p53βEGFP. Statistical comparisons made to control. Results are mean ± SD. *P < 0.05, ** P < 0.01.

**Supplementary Figure 3.** Excel spreadsheet will all the raw protein measurements from the mass spectrometry analyses.

**Supplementary Figure 4.** Heatmaps showing proteins increased or decreased on the Δ122p53 cell surface. Cell surface proteins were biotinylated, biotinylated proteins separated and identified using mass spectrometry. Graphics produced using ShinyGO 0.77(6). Log2-transformed protein abundance ratios of Δ122p53/ control cells line for B16F10, PDAC and MEF cell lines. 1, Experiment 1; 2, Experiment 2; 3, Experiment 3.

**Supplementary Figure 5.** Example R studio code for creating heatmaps of proteins increased and decreased from the mass spectrometry data.

**References**

1. Silva, J.C.*, et al.* (2006) Absolute quantification of proteins by LCMSE: a virtue of parallel MS acquisition. *Mol Cell Proteomics*, **5**, 144-56.

2. Wickham, H., François, R., Henry, L., Müller, K., & Vaughan, D. (2023). *dplyr: A grammar of data manipulation* (Version 1.1.4) [R package]. CRAN. [https://CRAN.R-project.org/package=dplyr](https://cran.r-project.org/package=dplyr)

3. Wickham, H., Vaughan, D., & Girlich, M. (2023). *tidyr: Tidy messy data* (Version 1.3.0) [R package]. CRAN. [https://CRAN.R-project.org/package=tidyr](https://cran.r-project.org/package=tidyr)

4. Warnes, G. R., Bolker, B., Bonebakker, L., Gentleman, R., Huber, W., Liaw, A., Lumley, T., Maechler, M., Magnusson, A., Moeller, S., Schwartz, M., & Venables, B. (2024). *gplots: Various R programming tools for plotting data* (Version 3.1.3.1) [R package]. CRAN. [https://CRAN.R-project.org/package=gplots](https://cran.r-project.org/package=gplots)

5. Neuwirth, E. (2022). *RColorBrewer: ColorBrewer palettes* (Version 1.1-3) [R package]. CRAN. [https://CRAN.R-project.org/package=RColorBrewer](https://cran.r-project.org/package=RColorBrewer)

6. Ge, S.X.*, et al.* (2020) ShinyGO: a graphical gene-set enrichment tool for animals and plants. *Bioinformatics*, **36**, 2628-2629.

**Supplementary Table 1. Antibodies and optimized conditions for immunostaining and western blotting analyses.**

| **Antibody** | **Application** | **Clone** | **Dilution** | **Antigen retrieval method** | **Manufacturer** |
| --- | --- | --- | --- | --- | --- |
| a-tubulin | WB | DM1A | 1/10000 IHC | - | Abcam |
| Biotin | WB | Rabbit polyclonal | 1/1000  IHC | - | Abcam |
| CD163 | IHC | EPR19518 | 1/600 | ER1, 20 minutes | Abcam |
| CD3 | IHC | Polyclonal | 1/400  IHC | ER1, 20 minutes | Abcam |
| Histone H3 | WB | EPR17785 | 1/5000  WB | - | Abcam |
| Ki67 | IHC on murine cells/tissues | SP6 | 1/200 IHC | ER2, 20 minutes | Abcam |
| Ki67 | IHC on human cells/tissues | MIB-1 | 1/100 IHC | ER2, 20 minutes | Dako, Agilent |
| pMAPK (p38) | IHC, IF | Thr180/Tyr182  (D3F9) | 1/800  IHC | ER1, 20 minutes | Cell Signaling |
| MelanA | IHC | EPR20380 | 1/50  IHC | ER1, 20 minutes | Abcam |
| PD1 | IHC | EPR20665 | 1/50  IHC | ER1, 20 minutes | Abcam |
| PDL1 | IF, human cell lines and tissues | CAL10 | 1/800  IHC, IF | ER1, 20 minutes | Biocare Medical |
| P53 PAb421 | IHC, IF, WB for human cell lines | BML-SA293-0050 | 1/100 IF  1/500 WB | ER1, 20 minutes | Enzo Life Sciences |
| P53 PAb421 | For murine cell lines and tissues | MABE283 | 1/500 WB | ER1, 20 minutes | Sigma Aldrich |
| P53 | IF, human cell lines | DO11 | 1/50 IF  1/200 WB | ER2, 20 minutes | Bio-rad laboratories |
| NF-kB p65 | IF, IHC | (D14E12) | 1/50  IHC, IF | ER2, 20 minutes | Cell signaling |

IHC, immunohistochemistry; IF, immunofluorescence; WB, western blotting

**Supplementary Table 2. Antibodies used for flow cytometry analyses.**

| **Antibody reactivity** | **Clone** | **Dilution** | **Manufacturer** |
| --- | --- | --- | --- |
| **ADAM10 (CD156c)** (human) | SHM14 (PE) | 1/100 | Biolegend |
| **AXL** (human) | S21005B (PE) | 1/50 | Biolegend |
| **API5** (human and mouse) | Polyclonal (PE) | 1/50 | Thermo Fisher Scientific |
| **CTPS1** (human) | JE63-85 (FITC secondary) | 1/100 | Thermo Fisher Scientific |
| **PLXNB2** (human) | FAB53291 (PE) | 1/100 | R and D Systems |
