# Supplementary material for: Δ133p53 isoform enhances TLR4 function to promote tumor growth
Source: Carcinogenesis. 2025 Aug 29;46(4):bgaf051. doi: 10.1093/carcin/bgaf051 (PMC12683340; doi:10.1093/carcin/bgaf051)

TumourSyngraftHeatmaps

Load in all data

```
B16_1_heat_label <- read.csv("B16_E1_Manual_Heat.csv")
row.names(B16_1_heat_label) <- make.unique((B16_1_heat_label$X))


B16_2_heat_label <- read.csv("B16_E2_Manual_Heat.csv")
row.names(B16_2_heat_label) <- make.unique((B16_2_heat_label$X))

B16_3_heat_label <- read.csv("B16_E3_Manual_Heatmap.csv")
row.names(B16_3_heat_label) <- make.unique((B16_3_heat_label$X))

PDAC_1_heat_label <- read.csv("PDAC_E1_Manual_Heat.csv")
row.names(PDAC_1_heat_label) <- make.unique((PDAC_1_heat_label$X))

PDAC_2_heat_label <- read.csv("PDAC_E2_Manual_Heat.csv")
row.names(PDAC_2_heat_label) <- make.unique((PDAC_2_heat_label$X))

PDAC_3_heat_label <- read.csv("PDAC_E3_Manual_Heat.csv")
row.names(PDAC_3_heat_label) <- make.unique((PDAC_3_heat_label$X))

MEF_1_heat_label <- read.csv("MEF_1_heat_labels.csv")
row.names(MEF_1_heat_label) <- MEF_1_heat_label$X
MEF_1_heat_label[MEF_1_heat_label == "#DIV/0!"] <- NA
MEF_1_heat_label <- na.omit(MEF_1_heat_label)




MEF_2_heat_label <- read.csv("MEF_2_heat_labels.csv")
row.names(MEF_2_heat_label) <- MEF_2_heat_label$X
MEF_2_heat_label[MEF_2_heat_label == "#DIV/0!"] <- NA
MEF_2_heat_label <- na.omit(MEF_2_heat_label)

MEF_3_heat_label <- read.csv("MEF_3_heat_labels.csv")
row.names(MEF_3_heat_label) <- MEF_3_heat_label$X
MEF_3_heat_label[MEF_3_heat_label == "#DIV/0!"] <- NA
MEF_3_heat_label <- na.omit(MEF_3_heat_label)

B16_up <- read.csv("B16_up.csv")
B16_down <- read.csv("B16_down.csv")
PDAC_up <- read.csv("PDAC_up.csv")
PDAC_down <- read.csv("PDAC_down.csv")

B16_up_genelist <- read.csv("B16_up_genelist.csv", header = FALSE)
B16_up_genelist$V1 <- sub("^\\s+", "", B16_up_genelist$V1)

B16_down_genelist <- read.csv("B16_down_genelist.csv", header = FALSE)
B16_down_genelist$V1 <- sub("^\\s+", "", B16_down_genelist$V1)

PDAC_up_genelist <- read.csv("PDAC_up_genelist.csv", header = FALSE)
PDAC_up_genelist$V1 <- sub("^\\s+", "", PDAC_up_genelist$V1)

PDAC_down_genelist <- read.csv("PDAC_down_genelist.csv", header = FALSE)
PDAC_down_genelist$V1 <- sub("^\\s+", "", PDAC_down_genelist$V1)

MEF_up_genelist <- read.csv("MEF_up_genelist.csv", header = FALSE)

MEF_down_genelist <- read.csv("MEF_down_genelist.csv", header = FALSE)
```

B16

```
B16_Manual <- merge(B16_1_heat_label, B16_2_heat_label, by = 'row.names', all = TRUE)
row.names(B16_Manual) <- B16_Manual$Row.names
B16_Manual <- B16_Manual %>%  dplyr::select(contains("AB"))

B16_Manual <- merge(B16_Manual, B16_3_heat_label, by = 'row.names', all = TRUE)
row.names(B16_Manual) <- B16_Manual$Row.names
B16_Manual <- B16_Manual %>%  dplyr::select(contains("AB"))
B16_Manual <- B16_Manual %>%  dplyr::select(contains("AB"))
## Remove less signif Tmpo
B16_Manual <- B16_Manual[!(row.names(B16_Manual) == "Tmpo"), ]
B16_Manual[B16_Manual == "#DIV/0!"] <- NA


for (i in 1:nrow(B16_up)) {
  old_name <- B16_up$old[i]
  new_name <- B16_up$new[i]

  # Check if the old_name exists as a row name in B16_Manual
  if (old_name %in% rownames(B16_Manual)) {
    # Find the row index of the old_name in B16_Manual
    row_index <- which(rownames(B16_Manual) == old_name)
    # Set the new_name as the row name in B16_Manual
    rownames(B16_Manual)[row_index] <- new_name
  }
}

for (i in 1:nrow(B16_down)) {
  old_name <- B16_down$old[i]
  new_name <- B16_down$new[i]

  # Check if the old_name exists as a row name in B16_Manual
  if (old_name %in% rownames(B16_Manual)) {
    # Find the row index of the old_name in B16_Manual
    row_index <- which(rownames(B16_Manual) == old_name)
    # Set the new_name as the row name in B16_Manual
    rownames(B16_Manual)[row_index] <- new_name
  }
}


rownames(B16_Manual) <- sub("^\\s+", "", rownames(B16_Manual))


B16_Manual_up <- B16_Manual[rownames(B16_Manual) %in% B16_up_genelist$V1, ]


B16_Manual_up[B16_Manual_up == 0] <- NA

B16_Manual_up_matrix <- (as.matrix(B16_Manual_up))
B16_Manual_up_matrix <- apply(B16_Manual_up_matrix, 2, as.numeric)
row.names(B16_Manual_up_matrix) <- row.names(B16_Manual_up)

B16_Manual_up_matrix <- log2(B16_Manual_up_matrix)

B16_Manual_up_Heat <- Heatmap(B16_Manual_up_matrix,
                 show_row_dend = FALSE,
                 show_column_dend = FALSE,
                 cluster_rows = FALSE,
                 cluster_columns = FALSE,
                 na_col = "black",
                 row_names_gp = gpar(fontsize = 2.75),
                name = "Log2 Abundance Ratio",
                col = (colorRamp2(c(-2, 0, 2), c("blue", "white", "red"))))
draw(B16_Manual_up_Heat)
```

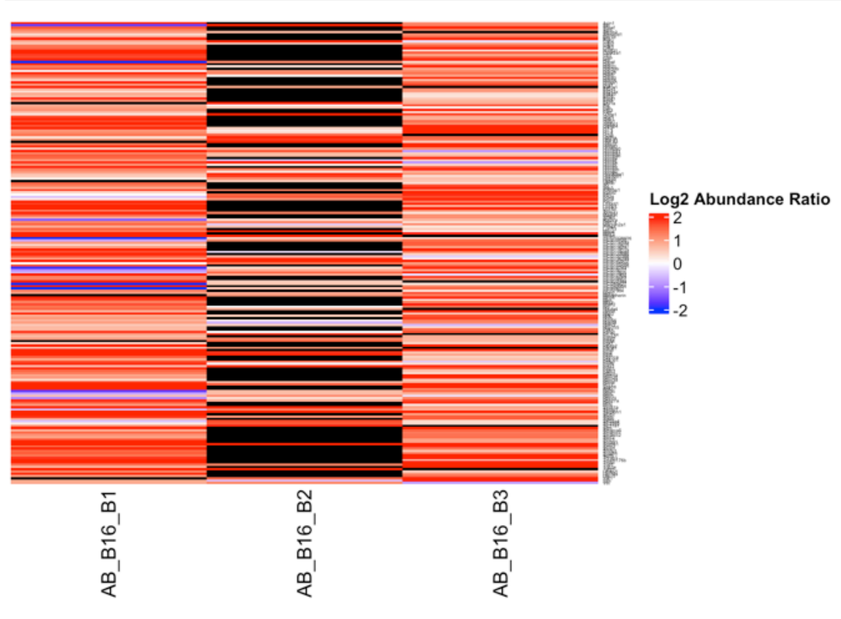


```
B16_Manual_down <- B16_Manual[rownames(B16_Manual) %in% B16_down_genelist$V1, ]

B16_Manual_down[B16_Manual_down == 0] <- NA

B16_Manual_down_matrix<- as.matrix(B16_Manual_down)
B16_Manual_down_matrix <- apply(B16_Manual_down_matrix, 2, as.numeric)
row.names(B16_Manual_down_matrix) <- row.names(B16_Manual_down)


B16_Manual_down_matrix <- log2(B16_Manual_down_matrix)

B16_Manual_down_Heat <- Heatmap(B16_Manual_down_matrix,
                           show_row_dend = FALSE,
                           show_column_dend = FALSE,
                           cluster_rows = FALSE,
                           cluster_columns = FALSE,
                           na_col = "black",
                           row_names_gp = gpar(fontsize = 4),
                           name = "Log2 Abundance Ratio",
                           col = (colorRamp2(c(-2, 0, 2), c("blue", "white", "red"))))
draw(B16_Manual_down_Heat)
```

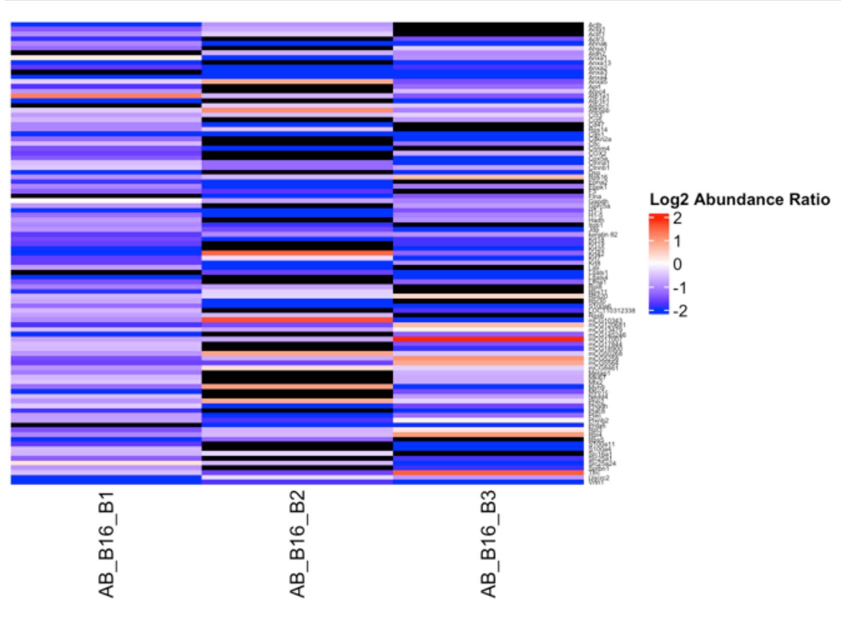

PDAC

```
PDAC_Manual <- merge(PDAC_1_heat_label, PDAC_2_heat_label, by = 'row.names', all = TRUE)
row.names(PDAC_Manual) <- PDAC_Manual$Row.names
PDAC_Manual <- PDAC_Manual %>%  dplyr::select(contains("AB"))

PDAC_Manual <- merge(PDAC_Manual, PDAC_3_heat_label, by = 'row.names', all = TRUE)
row.names(PDAC_Manual) <- PDAC_Manual$Row.names
PDAC_Manual <- PDAC_Manual %>%  dplyr::select(contains("AB"))

PDAC_Manual[PDAC_Manual == "#DIV/0!"] <- NA

for (i in 1:nrow(PDAC_up)) {
  old_name <- PDAC_up$old[i]
  new_name <- PDAC_up$new[i]

  # Check if the old_name exists as a row name in PDAC_Manual
  if (old_name %in% rownames(PDAC_Manual)) {
    # Find the row index of the old_name in PDAC_Manual
    row_index <- which(rownames(PDAC_Manual) == old_name)
    # Set the new_name as the row name in PDAC_Manual
    rownames(PDAC_Manual)[row_index] <- new_name
  }
}

for (i in 1:nrow(PDAC_down)) {
  old_name <- PDAC_down$old[i]
  new_name <- PDAC_down$new[i]

  # Check if the old_name exists as a row name in PDAC_Manual
  if (old_name %in% rownames(PDAC_Manual)) {
    # Find the row index of the old_name in PDAC_Manual
    row_index <- which(rownames(PDAC_Manual) == old_name)
    # Set the new_name as the row name in PDAC_Manual
    rownames(PDAC_Manual)[row_index] <- new_name
  }
}


rownames(PDAC_Manual) <- sub("^\\s+", "", rownames(PDAC_Manual))


PDAC_Manual_up <- PDAC_Manual[rownames(PDAC_Manual) %in% PDAC_up_genelist$V1, ]

PDAC_Manual_up[PDAC_Manual_up == 0] <- NA

PDAC_Manual_up_matrix<- as.matrix(PDAC_Manual_up)
PDAC_Manual_up_matrix <- apply(PDAC_Manual_up_matrix, 2, as.numeric)
row.names(PDAC_Manual_up_matrix) <- row.names(PDAC_Manual_up)


PDAC_Manual_up_matrix <- log2(PDAC_Manual_up_matrix)

PDAC_Manual_up_Heat <- Heatmap(PDAC_Manual_up_matrix,
                              show_row_dend = FALSE,
                              show_column_dend = FALSE,
                              cluster_rows = FALSE,
                              cluster_columns = FALSE,
                              na_col = "black",
                              row_names_gp = gpar(fontsize = 4),
                              name = "PDAC up",
                              col = (colorRamp2(c(-2, 0, 2), c("blue", "white", "red"))))
draw(PDAC_Manual_up_Heat)
```

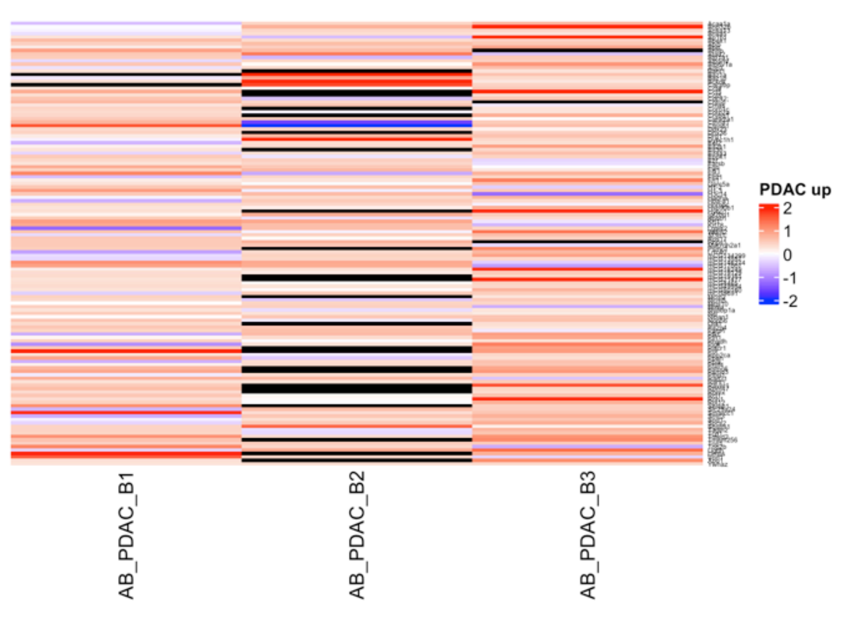


```
PDAC_Manual_down <- PDAC_Manual[rownames(PDAC_Manual) %in% PDAC_down_genelist$V1, ]

PDAC_Manual_down[PDAC_Manual_down == 0] <- NA

PDAC_Manual_down_matrix<- as.matrix(PDAC_Manual_down)
PDAC_Manual_down_matrix <- apply(PDAC_Manual_down_matrix, 2, as.numeric)
row.names(PDAC_Manual_down_matrix) <- row.names(PDAC_Manual_down)

PDAC_Manual_down_matrix <- log2(PDAC_Manual_down_matrix)

PDAC_Manual_down_Heat <- Heatmap(PDAC_Manual_down_matrix,
                                show_row_dend = FALSE,
                                show_column_dend = FALSE,
                                cluster_rows = FALSE,
                                cluster_columns = FALSE,
                                na_col = "black",
                                row_names_gp = gpar(fontsize = 4),
                                name = "PDAC down",
                                col = (colorRamp2(c(-2, 0, 2), c("blue", "white", "red"))))
draw(PDAC_Manual_down_Heat)
```

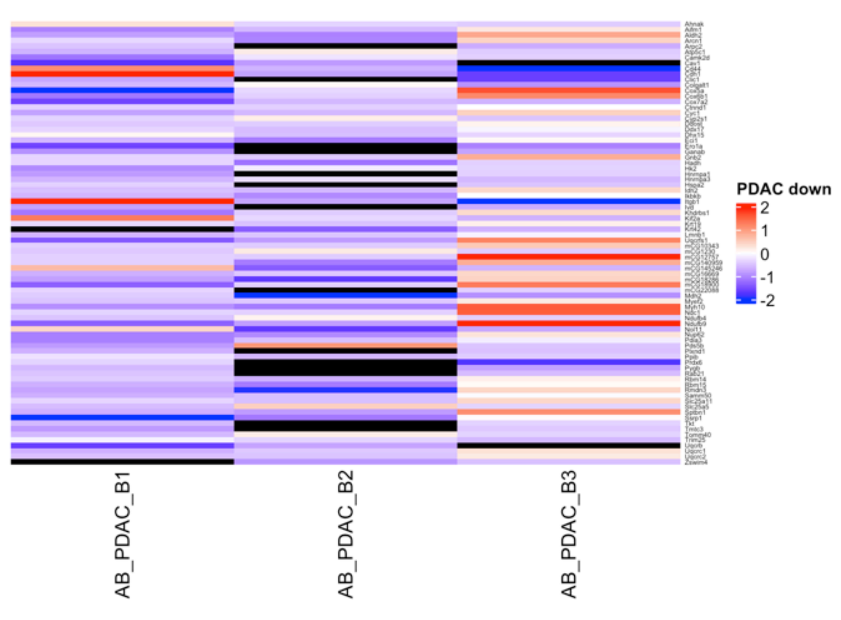

MEF

```
MEF_Manual <- merge(MEF_1_heat_label, MEF_2_heat_label, by = 'row.names', all = TRUE)
row.names(MEF_Manual) <- MEF_Manual$Row.names
MEF_Manual <- MEF_Manual %>%  dplyr::select(contains("AB"))

MEF_Manual <- merge(MEF_Manual, MEF_3_heat_label, by = 'row.names', all = TRUE)
row.names(MEF_Manual) <- MEF_Manual$Row.names
MEF_Manual <- MEF_Manual %>%  dplyr::select(contains("AB"))

MEF_Manual <- as.data.frame(MEF_Manual)

MEF_Manual_up <- MEF_Manual[rownames(MEF_Manual) %in% MEF_up_genelist$V1, ]

MEF_Manual_up_matrix <- as.matrix(MEF_Manual_up)
MEF_Manual_up_matrix <- apply(MEF_Manual_up_matrix, 2, as.numeric)
row.names(MEF_Manual_up_matrix) <- row.names(MEF_Manual_up)

MEF_Manual_up_matrix[MEF_Manual_up_matrix == 0.00] <- NA
MEF_Manual_up_matrix <- MEF_Manual_up_matrix[!(row.names(MEF_Manual_up_matrix) == "Hnrnpa3_Q0VG47"), ]
MEF_Manual_up_matrix <- MEF_Manual_up_matrix[!(row.names(MEF_Manual_up_matrix) == "Immt_Q3U7N2"), ]
MEF_Manual_up_matrix <- MEF_Manual_up_matrix[!(row.names(MEF_Manual_up_matrix) == "Anxa6_Q3UDK4"), ]
MEF_Manual_up_matrix <- MEF_Manual_up_matrix[!(row.names(MEF_Manual_up_matrix) == "Plec_Q05CR3"), ]
MEF_Manual_up_matrix <- MEF_Manual_up_matrix[!(row.names(MEF_Manual_up_matrix) == "Rps2_Q3TXS9"), ]
MEF_Manual_up_matrix <- MEF_Manual_up_matrix[!(row.names(MEF_Manual_up_matrix) == "Tbl3_A0JLN6"), ]
MEF_Manual_up_matrix <- MEF_Manual_up_matrix[!(row.names(MEF_Manual_up_matrix) == "Tmpo_Q61033"), ]
row.names(MEF_Manual_up_matrix) <- sub("_.*", "", row.names(MEF_Manual_up_matrix))



MEF_Manual_up_matrix <- log2(MEF_Manual_up_matrix)

MEF_Manual_up_Heat <- Heatmap(MEF_Manual_up_matrix,
                             show_row_dend = FALSE,
                             show_column_dend = FALSE,
                             cluster_rows = FALSE,
                             cluster_columns = FALSE,
                             na_col = "black",
                             row_names_gp = gpar(fontsize = 1.1),
                             name = "MEF up",
                             col = (colorRamp2(c(-2, 0, 2), c("blue", "white", "red"))))
draw(MEF_Manual_up_Heat)
```

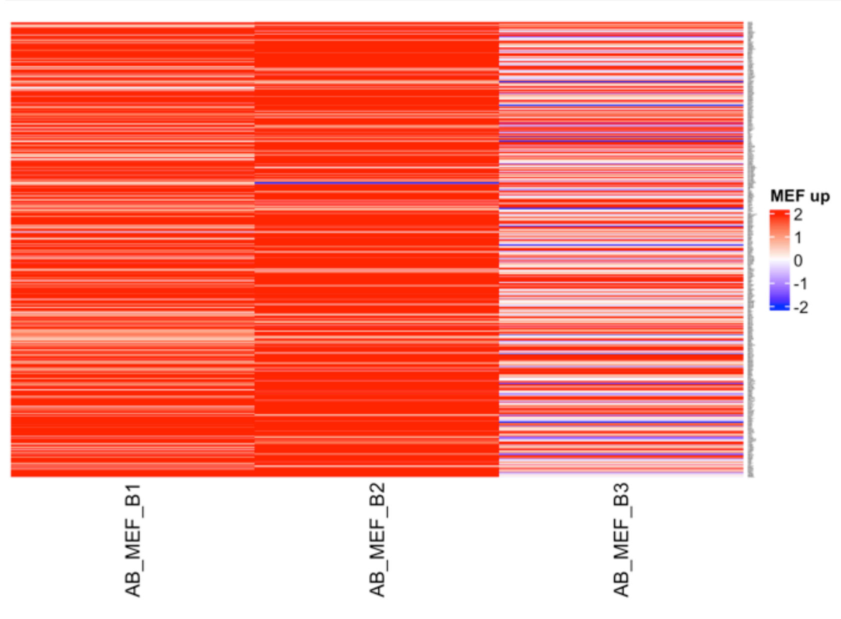


```
MEF_Manual_down <- MEF_Manual[rownames(MEF_Manual) %in% MEF_down_genelist$V1, ]

MEF_Manual_down_matrix <- as.matrix(MEF_Manual_down)
MEF_Manual_down_matrix <- apply(MEF_Manual_down_matrix, 2, as.numeric)
row.names(MEF_Manual_down_matrix) <- row.names(MEF_Manual_down)

MEF_Manual_down_matrix[MEF_Manual_down_matrix == 0.00] <- NA
MEF_Manual_down_matrix <- MEF_Manual_down_matrix[!(row.names(MEF_Manual_down_matrix) == "KRT1_Q61782"), ]
MEF_Manual_down_matrix <- MEF_Manual_down_matrix[!(row.names(MEF_Manual_down_matrix) == "PRSS1_Q9CPN9"), ]
row.names(MEF_Manual_down_matrix) <- sub("_.*", "", row.names(MEF_Manual_down_matrix))

MEF_Manual_down_matrix <- log2(MEF_Manual_down_matrix)

MEF_Manual_down_Heat <- Heatmap(MEF_Manual_down_matrix,
                               show_row_dend = FALSE,
                               show_column_dend = FALSE,
                               cluster_rows = FALSE,
                               cluster_columns = FALSE,
                               na_col = "black",
                               row_names_gp = gpar(fontsize = 4),
                               name = "MEF down",
                               col = (colorRamp2(c(-2, 0, 2), c("blue", "white", "red"))))
draw(MEF_Manual_down_Heat)
```

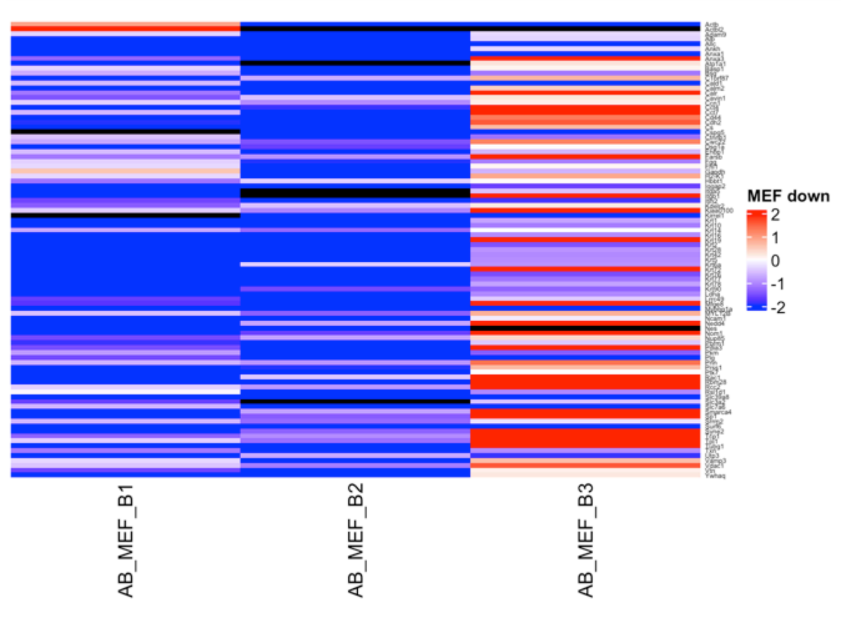

Supplement: bgaf051_Supplementary_Data [file bgaf051_supplementary_data.zip › Supplementary File 5.pdf]
