# Supplementary figures and images for: Δ133p53 isoform enhances TLR4 function to promote tumor growth
Source: Carcinogenesis. 2025 Aug 29;46(4):bgaf051. doi: 10.1093/carcin/bgaf051 (PMC12683340; doi:10.1093/carcin/bgaf051)

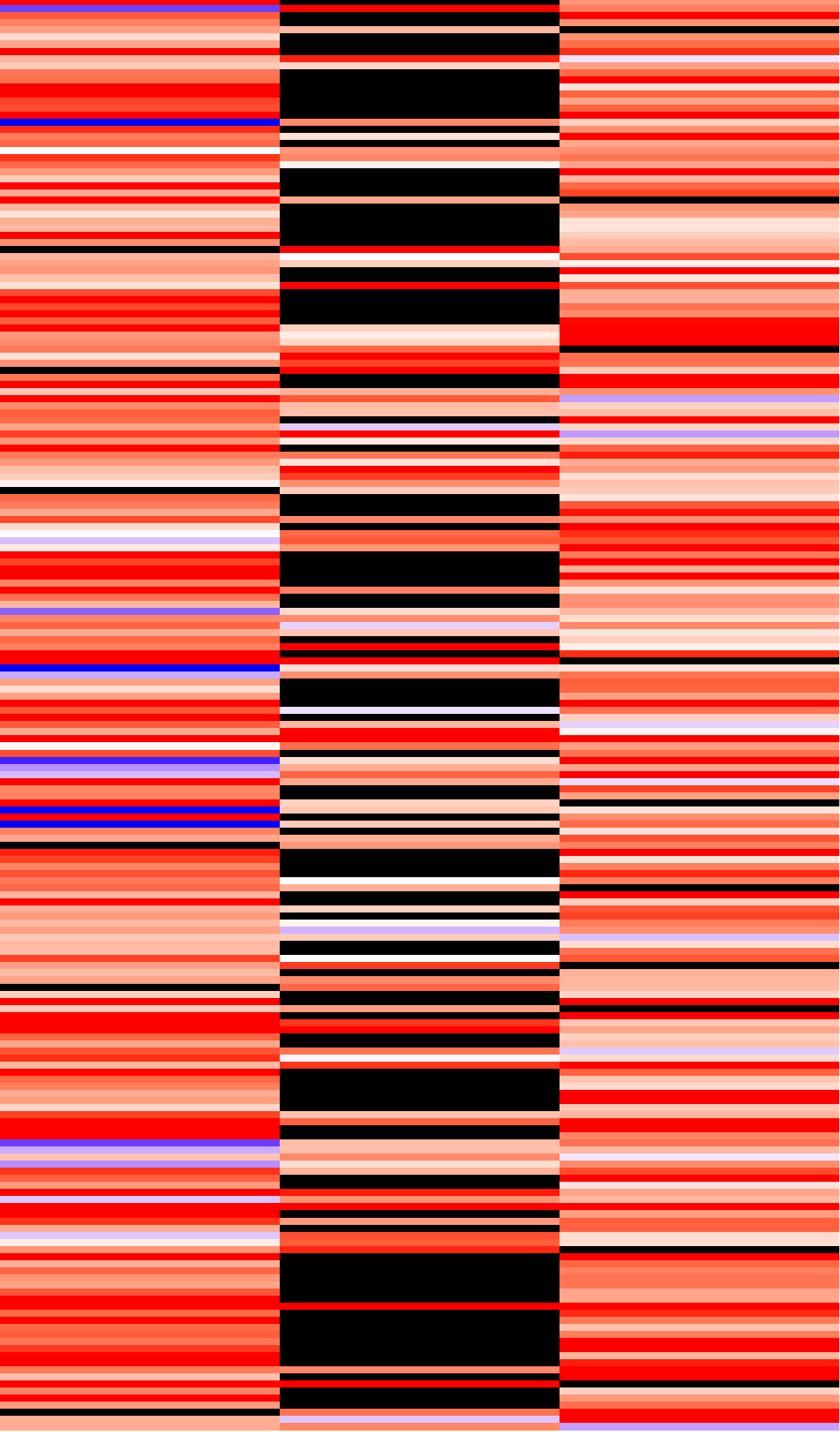

B16F10 increased

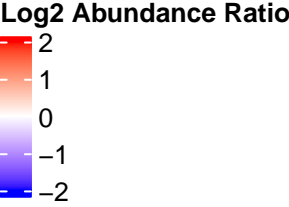

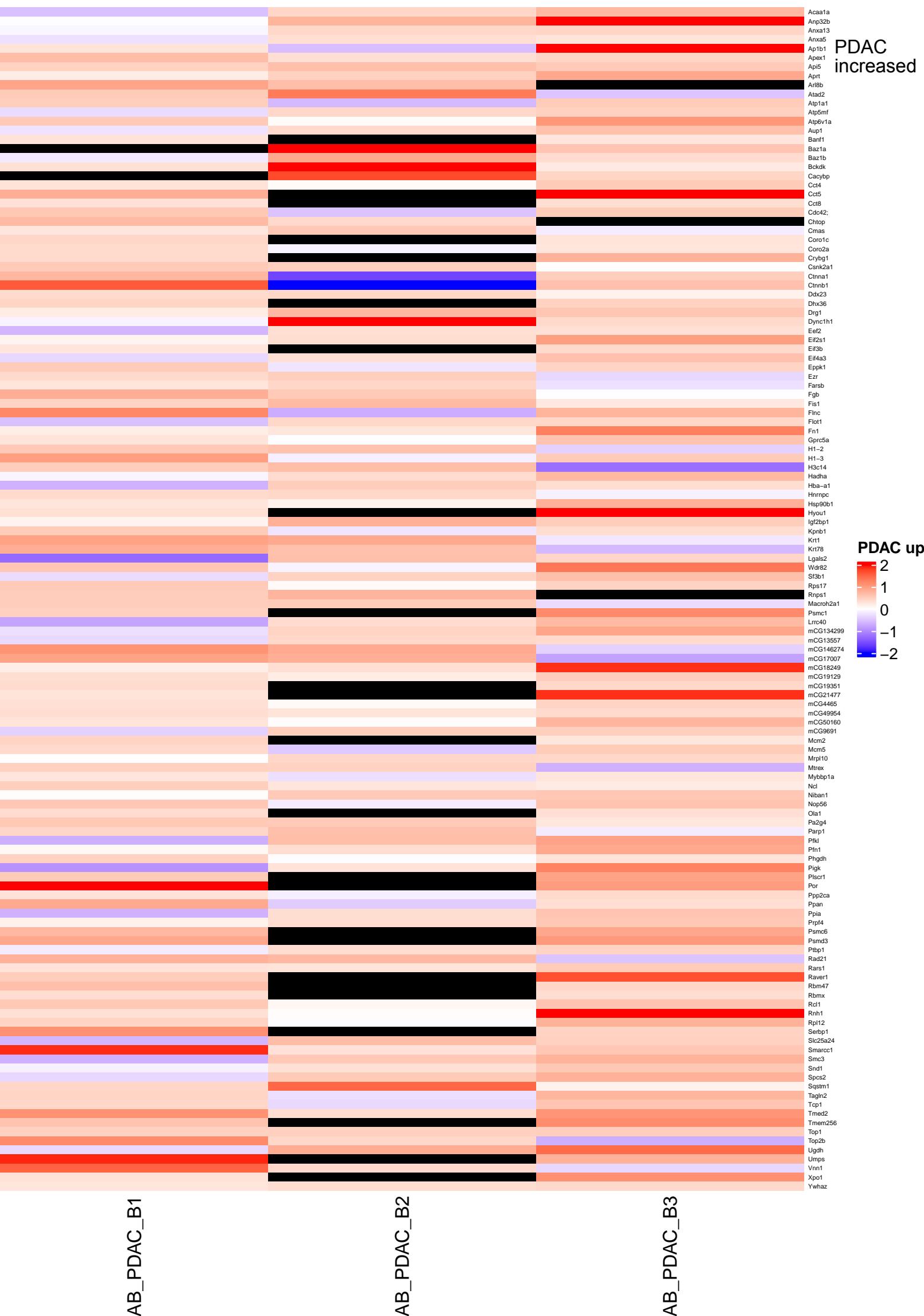

AB\_MEF\_B1

AB\_MEF\_B2

AB\_MEF\_B3

MEF up  
2  
1  
0  
-1  
-2

MEF  
increased

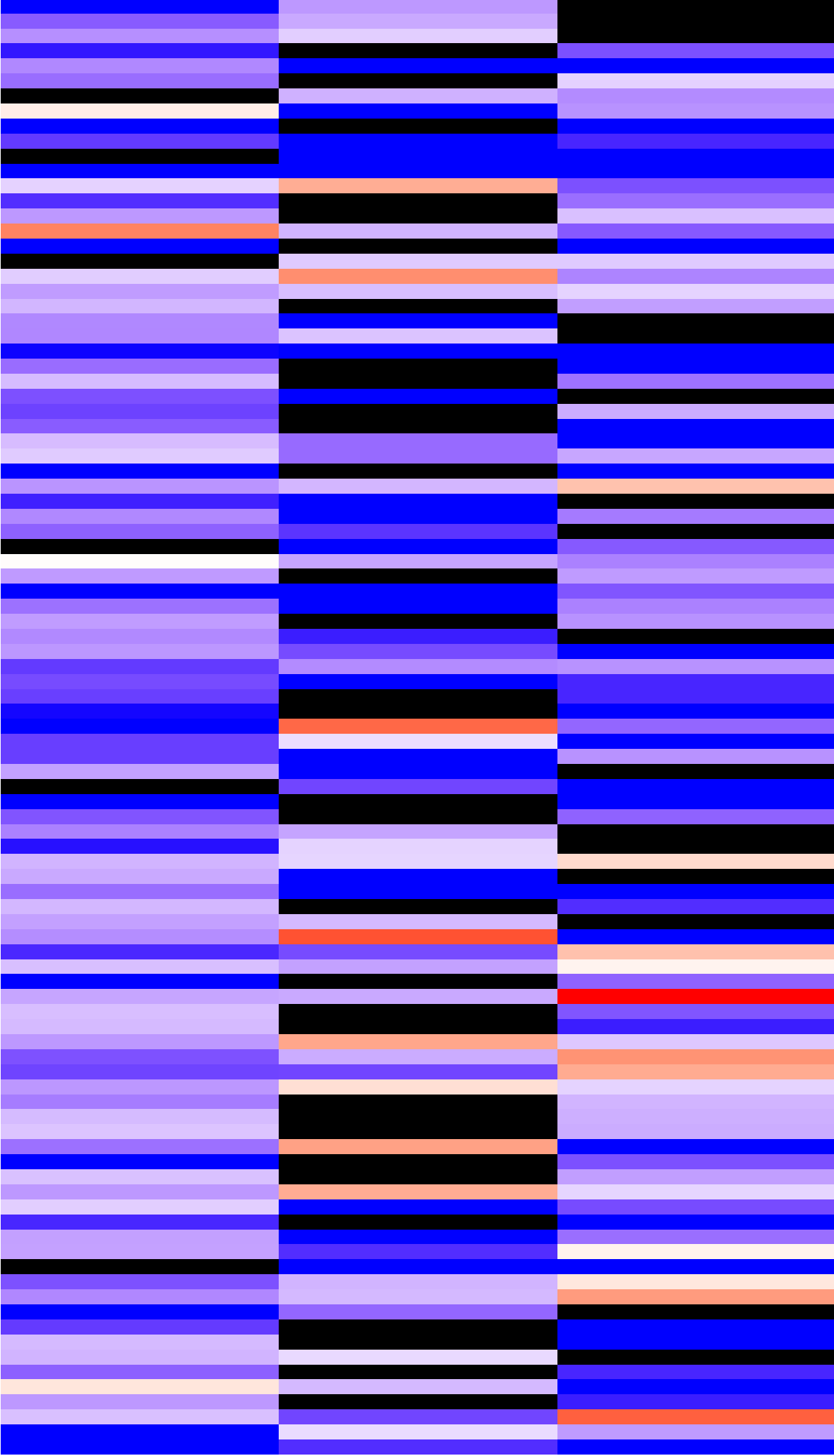

B16F10 decreased

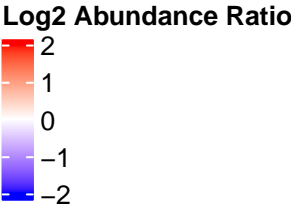

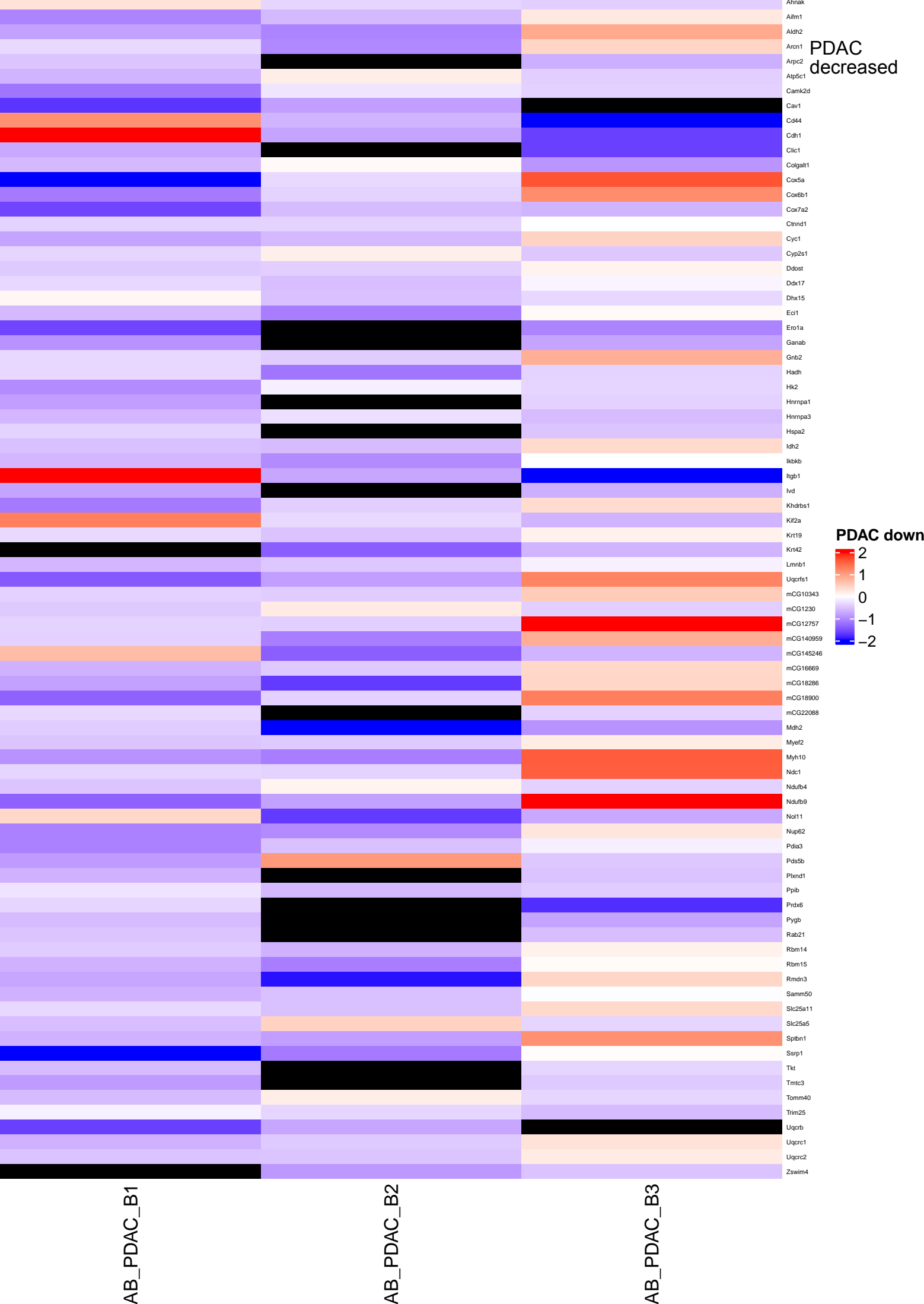

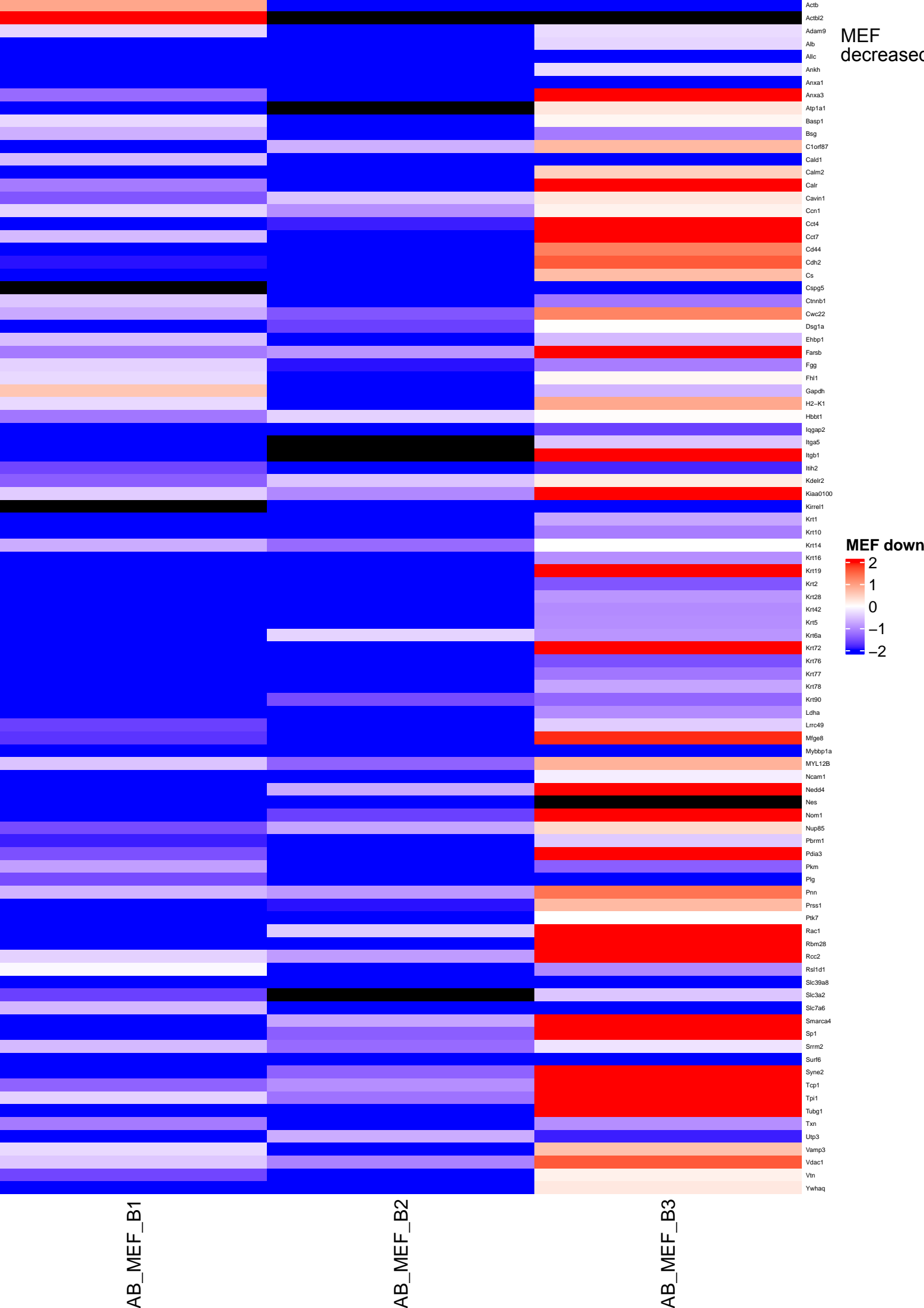

Supplement: bgaf051_Supplementary_Data [file bgaf051_supplementary_data.zip › Supplementary Figure 4.pdf]

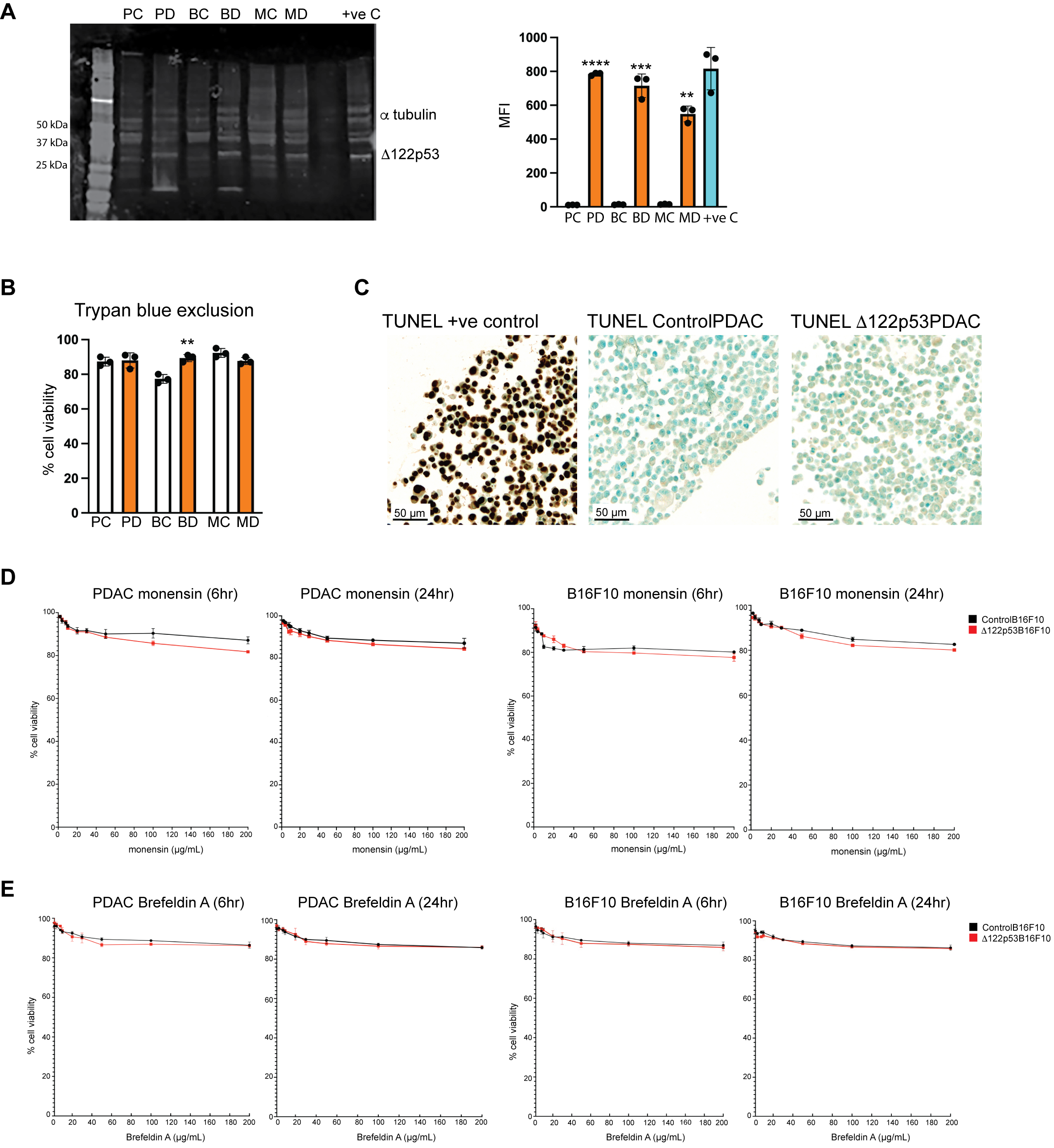

Supplement: bgaf051_Supplementary_Data [file bgaf051_supplementary_data.zip › Supplementary Figure 1 feb 2025.tif]

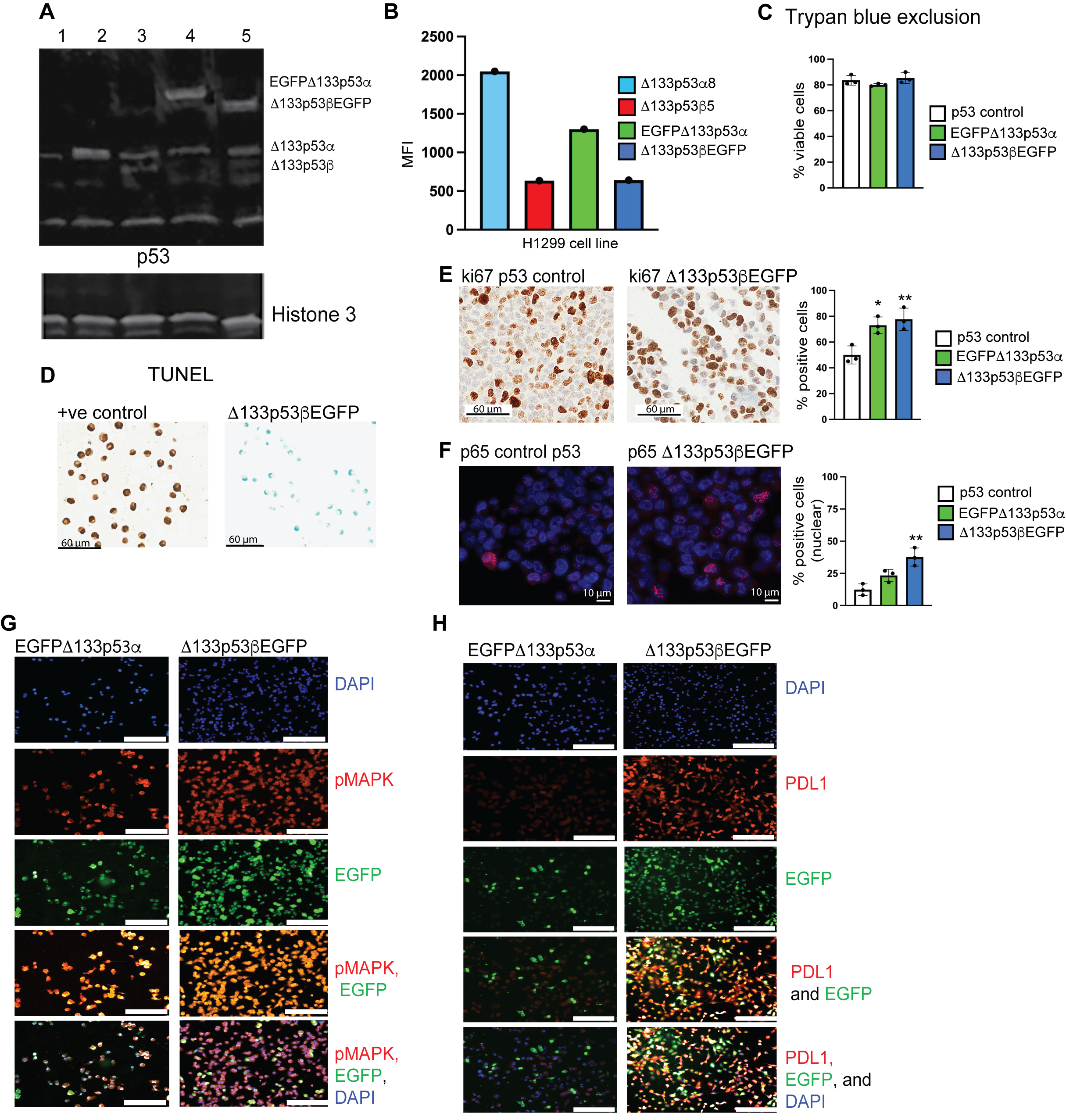

Supplement: bgaf051_Supplementary_Data [file bgaf051_supplementary_data.zip › Supplementary Figure 2 Feb 2025.tif]
